# Assessing cascading effects of climate change on the UK food system and food security

**DOI:** 10.1038/s41538-026-00990-6

**Published:** 2026-07-14

**Authors:** Sam Burkitt, Liza Jabbour, Taghi Miri, Helen Onyeaka

**Affiliations:** 1 Birmingham Institute for Sustainability and Climate Action, School of Chemical Engineering, University of Birmingham, Birmingham, UK; 2 Birmingham Institute for Sustainability and Climate Action, Birmingham Business School, University of Birmingham, Birmingham, UK

**Keywords:** Climate sciences, Environmental social sciences

## Abstract

This paper presents a holistic assessment of cascading climate risks on the security and resilience of food systems (FSs) in the United Kingdom (UK). It adopts a scoping review approach to assess vulnerabilities in the UK FS and to discuss methods used to develop cascading climate effect narratives. Results were synthesised to provide recommendations for building resilience and to guide future work. Import reliance and power imbalances are main sources of UK FS vulnerabilities. Climate change risks cascade through FSs and socio-economic systems, increase vulnerabilities and inequality and pose significant threat for food security. The findings highlight the need for greater standardisation in assessment approaches to produce a coherent body of research that can inform researchers, the general population and policy makers alike. The latter are particularly important due to their power to instigate the system level transformations required to safeguard food security in light of climate change.

## Introduction

Food systems (FSs), which link the production, processing, distribution and consumption of food, are under mounting strain. Malnutrition, widening socio-economic inequalities and ecosystem degradation already erode their capacity to deliver safe, nutritious diets^1–3^. Climate change adds a potent layer of risk; more frequent heatwaves, floods and droughts plus insidious shifts in temperature, rainfall and sea level undermine yields, weaken supply chain logistics and sow price volatility^4–6^. Together these pressures threaten every pillar of food security (availability, access, utilisation and stability) and, by extension, public health and sustainable development. Understanding how such hazards propagate through complex food chains is critical for designing resilient policies; this review addresses this need by examining the United Kingdom (UK) as a case study and synthesising evidence on cascading climate impacts, vulnerable nodes and feasible adaptation measures.

Whilst the effects of climate change on primary production are well documented, the implications for later stages in the food supply chain - such as reduced processing efficiency due to inconsistent quality and quantity of primary supply, negative impact on workers’ health and productivity due to extreme temperatures, disruptions to transport infrastructure as a result of extreme weather events (EWEs) and greater demand for refrigerated storage due to higher temperatures—are under-researched. These multi-faceted cascading effects (spanning physical, economic and social systems) underpin holistic food security^7,8^.

The UK is situated in a moderate temperature zone, experiencing cool summers and mild winters. However, longer term climate predictions project significant changes such as increased prevalence of floods, droughts and heat waves, mean temperature increases, changes to baseline conditions and increased climate variability through seasonal polarity, all of which have significant implications for agriculture, livestock and aquaculture^9–11^ (Supplementary Table 1). Although the UK is a high-income country with a relatively high level of food security^12^, a rise in the use of food-humanitarian aid, among other factors, indicates a steady decline in households’ food security over the last decade. Such an underlying onset of insecurity leaves portions of the UK population increasingly vulnerable to unexpected shocks to FSs^13,14^.

A systematic assessment of the impact of climate change on food security across the various dimensions of the FS is a requirement to inform climate change adaptation policies and to build resilience. In this paper, we adopt a scoping review approach to address this challenge. The review is explicitly designed to map and characterise existing literature across two interconnected domains: (i) the structure and vulnerabilities of the UK food system (SR1), and (ii) the cascading effects of climate change across systems (SR2). Cascading effects are operationally defined as sequences of climate-related impacts in which an initial climate hazard triggers secondary and tertiary effects that propagate across interconnected components of the food system, often crossing sectoral, spatial or governance boundaries. These effects are characterised by interdependence, temporal propagation and amplification through system linkages, rather than by direct exposure alone^7,8^. Cascading effects are conceptually distinct from, though related to, other commonly used risk concepts. Indirect effects refer to impacts that occur away from the initial point of climate exposure but do not necessarily involve multi-stage propagation through system linkages. Compound risks describe situations in which multiple climate hazards or stressors occur simultaneously or sequentially, increasing overall risk, but without an explicit focus on transmission pathways^4^. By contrast, cascading effects emphasise the process through which impacts spread and interact across system components, making them particularly relevant for analysing food systems, which are highly interconnected and multi-scalar. This definition is used consistently throughout the paper to frame the identification, mapping and interpretation of climate-related risks within the food system. By bringing these bodies of literature together, the review provides a structured synthesis of how cascading climate risks may manifest within the UK food system.

Consistent with the aims of a scoping review, this study does not seek to evaluate intervention effectiveness or develop new theory. Rather, it maps evidence, identifies recurring themes and analytical approaches and highlights points of convergence across heterogeneous studies. Interpretation and recommendations are derived from patterns observed across the mapped literature and are intended to support policy-relevant reflection on how cascading climate risks can be more systematically incorporated into food system resilience and food security assessments.

## Results

After a process of screening against the inclusion and exclusion criteria, the scoping review resulted in a total of 64 publications, 31 associated with SR1 and 33 associated with SR2 (Supplementary Fig. 3). A broad division was observed in the literature on cascading climate change effects between publications assessing the effects cascading from climate shocks, and those stemming from slow-onset climate changes. The latter received less attention, perhaps due to the more pressing nature of EWEs^15,16^. The publications on the UK FS/Security ranged from those that adopted a broad overview to those with a narrower focus (Table 1).

**Table 1:** Thematic focus of UK FS/security scoping review

Focus	Main findings	References
Local Food Systems/Smallholders/Small-medium Enterprises (SMEs)	There is divergent framing of the role of local FS actors in building resilience. These actors have limited influence on the overall operations of the FS. Support for smallholders has the potential to enhance food security and improve productivity and economic outcomes. Improving investment in traceability systems within the food supply chain, particularly by SMEs, is important for ensuring food safety.	34,36,90,33
National Diet/Consumers Behaviour	Food insecurity in disadvantaged communities is a system-wide problem linked to socio-economic conditions and the affordability or access to healthy food options. A shift towards a healthier, more environmentally sustainable diet requires a move away from a focus on consumers’ responsibility and education, to instead the adoption of system-wide interventions.	19,91,35,18
Food System Challenges and Transformation	The vulnerabilities of the UK FS are associated with a significant reliance on imports and just-in-time supply chains, the prevalence of UPFs and a power imbalance within the system. A fragmented governance landscape reduces the effectiveness of policy measures and limits the scope for system-level transformations.	26,32,27,92,22,93,94,95,23
Food Security	The availability and affordability of food in the UK are affected by climate shocks, locally and globally, through geopolitical tensions and disruption to supply chains. Food insecurity is more prevalent among younger people, households on lower income and home renters. Insulating the UK FS from the volatility of global food markets is essential for building resilience.	17,28,96,97
Food Supply Chains	Safeguarding food security is dependent on the resilience of food supply chains. Adaptive strategies, such as supply chain agility or investment in technological solutions, as well as continuous monitoring are required to enhance resilience and maintain food security.	98,29,37,31,99
Climate Change Impacts and Adaptation Measures	Domestic food productivity is dependent on changing climate conditions and potential adaptation measures. Radical changes to food production may be needed to maintain yields and build crop resistance. Food security measures and adaptive solutions require long-term economic assessments of potential trade-offs.	40,30,100,39,38

### UK food system/security

The literature on UK food system and food security reveals a set of interconnected themes that structure how climate-related risks propagate through the system. Across studies, socio-economic inequality is consistently framed as a core driver of food insecurity, with affordability, access and diet quality acting as key amplifiers of risk. At the same time, the UK’s strong exposure to international and trans-boundary shocks through import dependence and optimised global supply chains increases vulnerability to climate, geopolitical and economic disruptions. These risks are further intensified by structural power imbalances within the food system, particularly the dominance of large retailers, which limits the influence of producers and local actors and shifts shocks onto more vulnerable groups. In parallel, fragmented governance and lack of systems thinking constrain the ability of policy interventions to address these challenges holistically, often resulting in disconnected responses that fail to account for feedback loops and cascading effects. Finally, the literature converges on system-level and technological pathways for enhancing resilience, including rebalancing power relations, strengthening local food systems and leveraging technological and data-driven solutions to improve coordination, traceability and anticipatory decision-making.

The literature consistently identifies socio-economic inequality as a central driver and amplifier of food insecurity in the UK. Domestic food production, historically focused on maximising calories per hectare, does not necessarily ensure equitable access to healthy diets, and rising numbers of food-insecure households highlight the limitations of current measures of success. Cascading pathways emerge as socio-economic pressures translate into food choices and exacerbates nutritional deficiencies and health risks. These inequalities interact with broader systemic pressures, contributing to a cascade in which economic constraints, dietary patterns and health outcomes reinforce one another.

Socio-economic factors are significant drivers of food insecurity^17^. In a context where food purchasing decisions are primarily driven by prices and financial pressures^18^, individuals on low-income resort to cheaper, lower quality and often ultra-processed food (UFP) options. The limited access^19^ to healthy, often more expensive^20^ options compounds these individuals’ food insecurity with higher health risks^21^. Equally as concerning, is the prevalence of UFPs across all categories of households. The UK consumes the third highest volume of UPF per capita out of 80 high-and middle-income countries globally and has the most processed diet in Europe, leading to an obesity epidemic, particularly among low-income households^22–25^. The ongoing rise in UK households classified as food insecure suggests the need for a new framing of what constitutes a measure of success, for example a move towards maximising the number of people receiving a healthy diet per hectare instead of maximising calories per hectare of land^26^. Moreover, existing efforts at solving inequalities have not extended beyond diet and health incentives for disadvantaged communities. Food insecurity, driven by socio-economic inequalities, is likely to become more prevalent as food prices are projected to increase in most future FS scenarios^27^.

The UK’s vulnerability to international shocks represents another recurring theme. Heavy reliance on imported food and highly optimised global supply chains increases exposure to climate events, geopolitical conflicts, pandemics and market fluctuations. These systemic vulnerabilities propagate through supply chains, resulting in domestic price volatility and reduced food availability. As a net importer in most food categories^22,28^, the UK significantly depends on global supply chains for its food supply^29,30^. This limits the country’s ability to fully control its food security, which will partially depend on the future direction of the global FS in terms of trade openness or protectionism.

The exposure to international shocks is amplified by the complex structure of global food supply chains. This complexity is due to a strong emphasis on optimisation^31^, which improves efficiency and financial gains, but also increases the risks associated with disruptions along the chain and amplifies rippling effects. Global challenges, including climate change, conflicts, pandemics and geopolitics, render global food security increasingly volatile^28,32^. Moreover, the complexity of global food chains raises traceability issues which are widely recognised by food chain actors but face a lack of consensus as to whose responsibility it is to invest in solutions^33^. This vulnerability is exacerbated by a significant degree of power imbalance within the UK FS which is dominated by a small number of very large enterprises, particularly at the retail stage. Producers and local actors have little influence over the operations of the wider system despite their vital role in increasing its resilience^34^. Instead of moving to more local systems, the economic drive of retailers has led to trade routes that while complex, lack diversity^28^, thereby increasing vulnerability to climate shocks. Retailers can avert the consequences of potential disruptions by increasing their food prices^35^ and shifting the burdens to producers and consumers.

A consistent finding in the literature is the *lack of system thinking* within the UK food system, which amplifies vulnerabilities and limits the effectiveness of interventions. While many resilience measures are implemented at specific stages of the system, they rarely influence risks faced by other actors, creating disconnected and sometimes counterproductive outcomes. The vulnerabilities within the UK FS are complex, multidimensional and require a systemic approach, however most interventions and solutions focus on singular aspects in isolation^29,35,36^. Even when measures, implemented at a given stage, are successful in building resilience, these actions rarely have an impact on the risks of other actors within the system^37^. The lack of system thinking is partially associated with the governance structure of the UK FS, which is overseen by over 16 bodies. Additionally, the government’s Department for Environmental, Food and Rural Affairs relies on over 30 bodies for its remits^32^. Aligning all of these bodies and defining their responsibilities is challenging, resulting in a disconnected policy landscape that hampers policymakers’ ability to tackle complex systemic issues and to predict and assess feedback loops^38,39^. The lack of coordination is further exacerbated by the effects climate change has across the system, as it is often a challenge to determine whose responsibility it is to fund and implement climate resilience measures^40^.

The review highlights a set of actions aimed at increasing food security (Table 2). Most prominent is the need to redress the power imbalance in favour of producers and local food systems, and to adopt system level solutions instead of disconnected approaches. Some solutions, such as the ones outlined in the National Food Strategy (NFS), suggest a systemic reshaping of the FS^41^. Although the NFS presents a roadmap towards a more resilient FS, it has received mixed responses from consumers and limited engagement from policymakers^23^. Other, more targeted solutions, include the digitisation of food supply chains, through the ‘Internet of Things’ as a way to enhance food traceability, reduce fraud and tackle food waste^31,42,43^ or extending weather forecasting models to encompass the food chain and improve decision making and planning^40^.

**Table 2:** Recommended actions to enhance resilience in the UK FS

Recommendations		Number of publications
System Level Approaches	Adopting dynamic and integrated approaches that involve whole-system interventions instead of disconnected measures. Recognising the contribution of diverse forms of expertise and the need for collaboration between stakeholders. Assessing the synergies and trade-offs associated with FS transformation. Favouring ‘win-win’ solutions that emphasise the co-benefits of climate adaptation.	*n* = 13
A Power-Balanced System	Addressing the power imbalance within the UK FS in a way that favours producers and local FSs. Investing in the development of short and local supply chains. Recognising the vital role of SMEs and smallholders in building resilience within the system. Ensuring that the cost of adaptation measures are spread equitably across the system.	*n* = 8
Building Resilience	Improving domestic production and increasing the yield of local primary food production to enhance self-sufficiency. Investing in technological solutions to increase the resistance of crops and livestock to changing climate conditions. Investing in the education and training of a new generation of agriculture workforce equipped with the skills required for transforming domestic food production. Leveraging advances in data sciences to better forecast the implications of climate change for the FS and to implement early earning systems.	*n* = 8
Resilient Supply Chains	Improving the management of complex food supply chains through digitalisation. Developing advanced technological solutions that go beyond financial considerations to embed holistic sustainability. Improving transport connectivity, diversifying trade routes for food imports and adopting agile supply chain systems.	*n* = 6
Prioritising Socio-Economic Factors	Shifting the framing of food security from the availability of food to the accessibility of a healthy diet. Transforming the FS in a way that addresses the issue of poor diet and the inequality of food security and health outcomes across the population.	*n* = 5
Dietary Changes	Encouraging a dietary shift to more climate resilient options, like plant-based food alternatives and away from unhealthy UPFs.	*n* = 3
Balanced Land-Use Policies	Embedding wider metrics of sustainability in land-use policy frameworks to achieve a balance between climate mitigation objectives, food security and human and planetary health. Prioritising FS transformations that reduce carbon emissions while also regenerating ecosystems, enhancing biodiversity, reducing inequalities and improving population health.	*n* = 3
Enhancing Traceability	Adopting ‘Internet of Things’ technologies to allow the development of a trustworthy digital network and therefore increase food traceability and safety. The deployment of such technological solutions require a comprehensive data sharing infrastructure and coordination between stakeholders across the supply chain.	*n* = 3

### Cascading effects of climate change

Climate risk propagates through socio-economic systems, the increasing complexity of which makes systemic effects difficult to predict^44^. Unforeseen risks arise from a wide range of factors such as the delayed sharing of information, regional differences in climate impacts and feedback loops between multiple direct effects^45^. Additionally, mitigation measures may in turn initiate another series of cascading effects^46^. As a result, despite a consensus on the nature of direct effects within specific systems, the way in which these will cascade varied greatly, with different studies generating significantly different narratives. For example, in the context of FSs, some studies observed agricultural losses initiating a divide in the water-energy-food nexus whilst others highlighted propagation pathways extending beyond food supply into a series of economic implications and losses^47,48^.

**Fig. 1:**
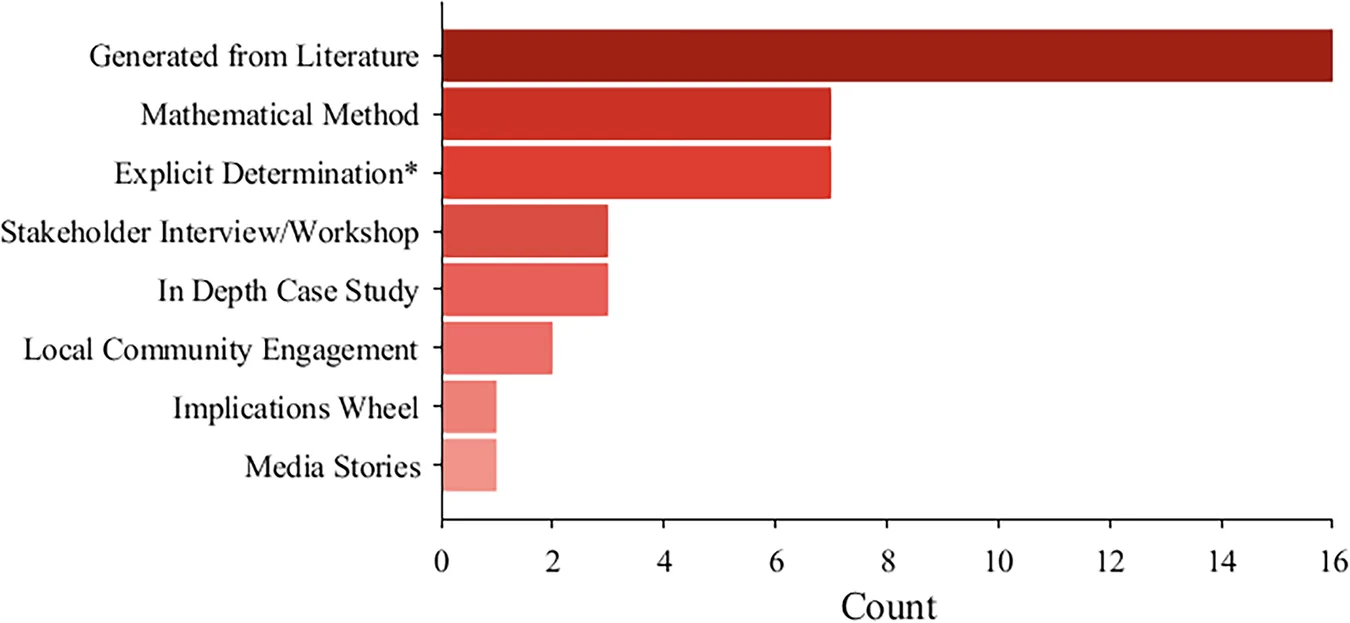
Approaches used to generate cascading effect narratives. Literature Analysis^16,38,47–49,58–60,63,69,81–86^; Explicit Determination(*via Experiments/Field Work)^36,51–55,64^; Predictive Modelling^36,38,45,48,83,87,88^; Stakeholders Engagement^15,49,50^; Case Studies^44,59,64^; Local Community Engagement^84,89^; Implications Wheel^58^, Media Stories^49^.

The significant variability in cascading effects narratives underscores the importance of the method used to identify these. However, the review shows that methods also varied greatly and lacked a standardised approach (Fig. 1). Beyond the most intuitive way of generating cascading narratives via piecing together effects seen in the literature, there was no methodological consensus.

The majority of papers relied on the existing literature to highlight existing cascading narratives or to illustrate new ones. The predominance of this approach is probably due to the relative ease and accessibility in which narratives can be generated, however the lack of standardisation in this approach casts doubt on the credibility of findings. Pulling together research ideas from different strands of literature, while straightforward, may not be the most robust methodological approach to generate cascading narratives if source papers consider a variety of contexts. Having said that, assessing the validity of this methodological approach is beyond the scope of this review and is a topic for future research.

Some studies explicitly developed narratives through direct experimentation, simulation or field work while others adopted a predictive modelling approach to forecast future effects. Only three publications consulted stakeholders with direct experience of the systems under consideration^47,49,50^. Whilst it is acknowledged that the capacity to consult stakeholders depends on the system/context in which cascading effects are being studied, it is worth noting the infrequency in which the direct experiences of impacted stakeholders are taken into account when generating cascading effects narratives.

The sub-group of publications that conducted lab experimentations, simulations or field work tended to focus on niche systems (e.g. soil microorganisms, plankton community structure, parasitism in animals)^51–53^, or on specific geographic ecosystems (e.g. subarctic lakes, alpine tree line dynamics)^54,55^. Whilst very specific and not directly relevant for the assessment of cascading climate effects within whole systems, such approaches could provide insight into how potential effects may be further explored or quantified.

The lack of methodological consensus made it difficult to compare narratives, highlight the most significant risks, identify commonalities in how effects cascaded through different systems and build a cohesive body of evidence that can inform policy.

**Fig. 2:**
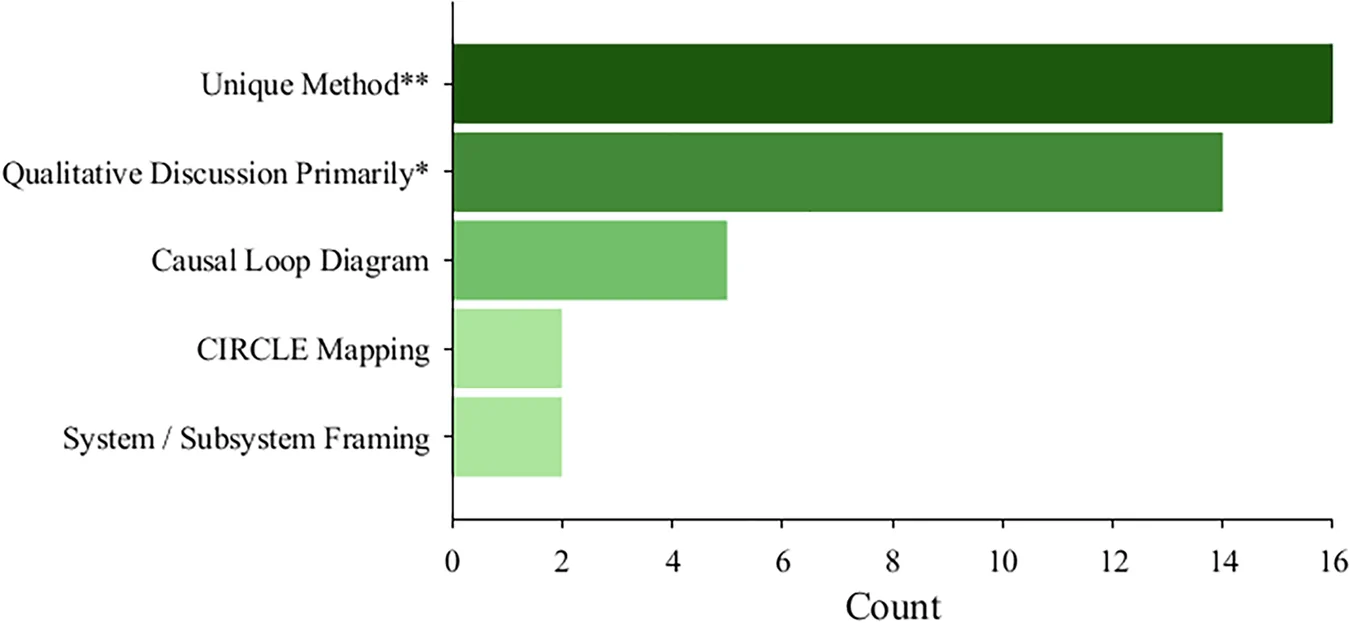
Approaches used to map cascading effect narratives. Unique Methods (**including original mapping-figures and using methodological approaches solely used in one paper)^15,44–46,50,52,55,58,60,63,64,82,84,85,88,89^; Qualitative Discussion(*Not limited to only qualitative discussion)^16,50–54,60,69,81–83,85–87^; Causal Loop Diagram^15,36,38,47,49^; CIRCLE Mapping^15,49^: System/SubSystem Framing^47,59^.

Similarly to the range in existing methods used to determine the cascading effects of climate shocks, there was also a wide variety of approaches taken to map and visually represent these effects (Fig. 2). Some papers generated original mapping approaches that were unique to their study while many others adopted a predominately qualitative discussion approach. In the case of the latter, publications often had complex, thought out processes by which cascading effects of climate change had been determined or predicted, but described the results through discussion with no formal mapping framework. Whilst informative, this approach made it difficult to draw comparisons and to identify common patterns in how climate change effects cascaded through the respective systems. Furthermore, the often-complex qualitative discussions may not very informative to policy makers and non-expert stakeholders who may have the power to implement adaptation and resilience measures.

**Fig. 3:**
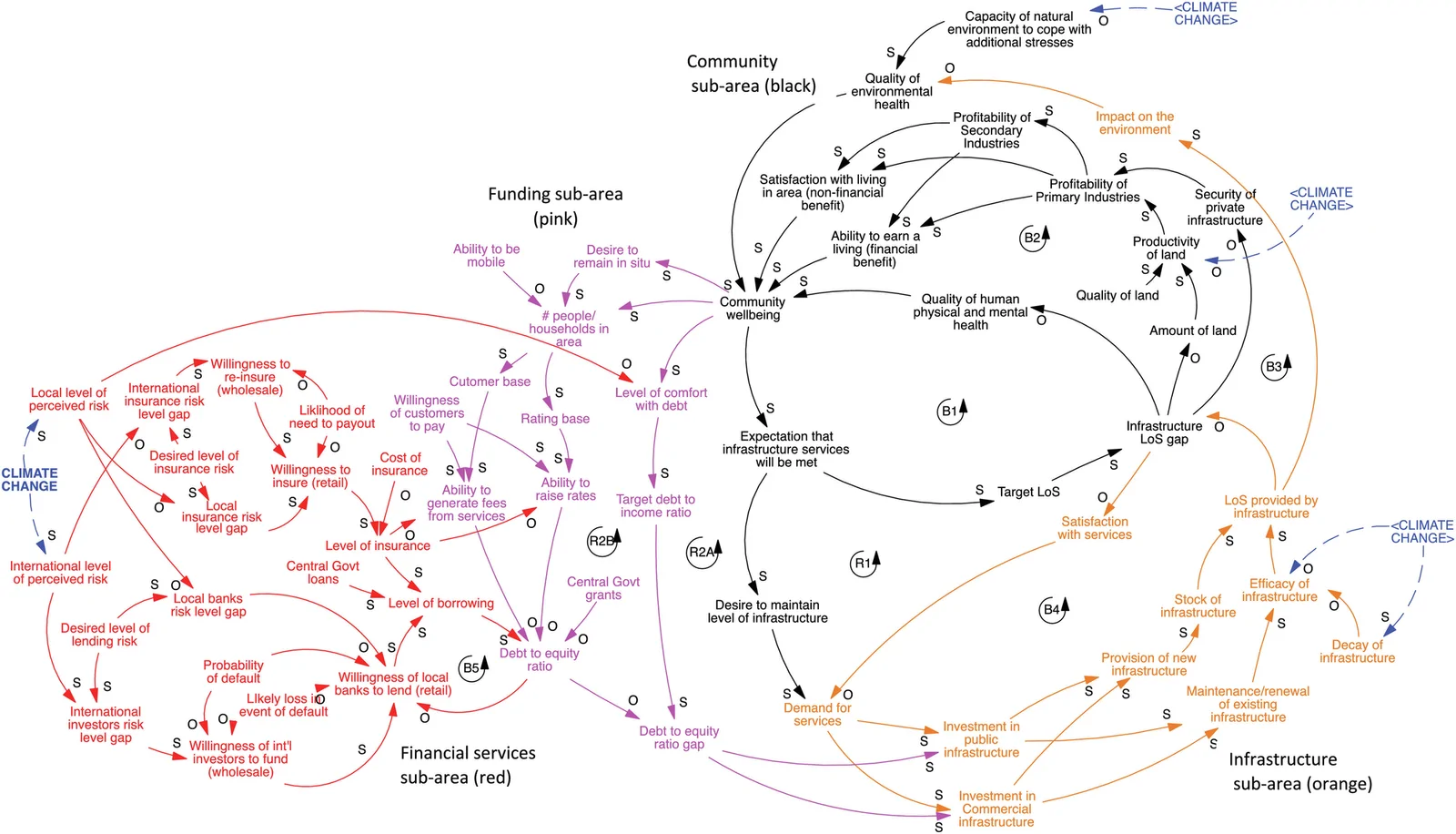
Example of a causal flow diagram framework. Example explores cascading climate impacts among all of ‘biophysical, physical, cultural, social, political, organisational and economic’ systems^49^.

The most repeated formal framework seen in the reviewed publications was Causal Loop Diagrams (*n* = 5). A Causal Loop Diagram (CLD) is a well-used tool in systems thinking which maps out the structure of, and feedbacks within, a system in order to understand its dynamics^56^ (Fig. 3). CLDs have the ability to go beyond visualisation to offer insights into the magnitude of effects. For example, a greater number of connections in a CLD implies potential for climate effects to be wider reaching, and a higher number of re-enforcing feedback loops indicates a more dynamic and unpredictable system^47^. This combined with the consistent format of the CLD meant it was the only method that facilitated direct comparison between publications.

Other methods used by multiple publications were the CIRCLE (Critical Infrastructure: Relations and Consequences for Life and the Environment) and system/subsystem analytical framework, each adopted by two publications (Supplementary Figs. 1 and 2). Both methods preceded the development of a CLD and thus were effective initial tools to assess the connections between the main system under consideration and its constituent sub-systems and infrastructure. Although methods such as CLDs, CIRLCE and system/subsystem framing are notable exceptions, a lack of standardisation regarding frameworks used to display and communicate cascading climate effects was evident. This again has negative implications for the assessment of cascading effects due to the limited ability to compare individual studies and to build a cohesive body of evidence that can inform policy.

The review emphasises the importance of adopting a holistic perspective on vulnerability (i.e. as a function of risk exposure, sensitivity and capacity to adapt^57^) as the same climate risks can produce dramatically different cascading effects due to variations in respective vulnerability^44,48,58,59^. Looking beyond direct effects is not sufficient to assess the magnitude of cascading risks. A linear model of consecutive effects conceals feedback loops within systems and misinforms policy makers, resulting in suboptimal responses^15,45,59–61^. This extends to impact assessment models, wherein single-sector models, which consider only one sector as opposed to the interaction between multiple sectors, were found to misrepresent the patterns and magnitudes of most climate impacts under study^62^.

A notable finding is the importance of effectively mapping a system; however, there was limited consensus as to what effective mapping should look like. Given the complexity of contemporary systems, identifying cascading effects requires both model-based quantification and qualitative assessment^45^. However, this is challenged by a lack of high-quality data^61^. Successfully visualising complex effects while ensuring a mapping technique is sufficiently accessible is another major challenge^63^ and perhaps a reason why the field has received relatively little attention.

The review emphasises the importance of early warning systems, continuous surveillance and improved forecasting. The meteorological nature of direct effects implies that effective active surveillance and connected early warning systems are potential adaptation strategies that would allow cascading effects to be predicted and resilience measures to be put in place ahead of time^60^. Scenario simulation and modelling software can be valuable tools that provide decision-makers with a comprehensible view of complex cascading effects^54,64^.

Finally, redressing socio-economic imbalances and considering social factors in policy making is required to reduce the overall consequences of cascading climate effects. The complexity of socio-economic systems leads to wider-reaching effects that increase overall systemic risk^44^. Environmental systems are strongly linked to socio-economic drivers and as such the highly uncertain future of many socio-economic systems make future cascading climate effects harder to predict. However, insufficient attention is currently being paid to social factors^62^, which represents a significant limitation of most impact assessment models^65^. Failing to take socio-economic factors into consideration may lead to unintended consequences and sub-optimal outcomes of adaptation policies, particularly in the global FS where socio-economic disparities are relatively significant^66–69^.

## Discussion

The assessment of cascading climate risks within FSs needs to take into account contextual vulnerabilities. In the UK, the reliance on imports exposes the FS to the direct effects of overseas climate events. Whilst domestic climate risks are predominantly confined to flooding, droughts, heatwaves and cold snaps, the global food supply chains will inevitably be subject to further climate hazards of varying nature, intensity and frequency. While direct climate effects are easier to predict, the wide variety of possible cascading effects from these are less so and cause greater systemic issues. Exposure to international climate hazards increases the scope of risks and expands the number of possible indirect challenges to the FS. Building resilience requires monitoring and modelling international climate hazards but faces significant barriers including the lack of traceability within complex global supply chains and delays in information sharing between countries. The UK could try to overcome these challenges by investing in early detections and using technological advances to enhance traceability and facilitate the modelling of overseas climate hazards. An alternative approach would be to prioritise local FSs and domestic production. Both approaches highlight the challenges posed by cascading climate risks and the system-wide interventions required to increase resilience.

A common theme across both reviews is the importance of socio-economic factors. Whilst most direct effects of climate change are biophysical and therefore impact bio-physical systems, often-times the resulting indirect effects act on, and propagate through, differences within socio-economic systems. In the context of the UK where income inequalities drive food insecurity, independently of the effects of climate change, the interplay between direct climate risks and socio-economic cascading pathways represents a significant threat to overall food security. The power imbalance among food supply chain actors and the lack of support for low income and disadvantaged households are root causes of contextual vulnerability and of the resulting food insecurity, therefore redressing these should be prioritised as resilience measures.

Another key conclusion from both reviews is the importance of systems thinking to both resilient FSs and resilience to cascading climate shocks. Single-sector technological innovations may mitigate direct climate shocks but do not eliminate systemic risks. In the UK context, land area is largely the limiting factor to the domestic production of key crops^39^. While technology may be able to increase this via improvements in soil arability improvements or increasing land area in other ways (i.e. vertical farming), wider-reaching solutions likely need to be socio-economic. For example, a more effective way of increasing the calorie efficiency of available land is drastically reducing meat production^70^, which will in-turn require a population dietary shift that depends on enabling policy measures and significant behavioural change. The reliance on imports also limits the potential of technological solutions as implementation in overseas production will likely be out of the UK’s direct control. As a result, whilst some resilience may be achieved by single-sector solutions, more systemic climate resilience may require combining socio-economic interventions with technological solutions when needed. However, the fragmented governance structure of the UK’s FS makes system-wide approaches difficult to implement. Effective changes would require agreement and close communication between the different governing bodies to ensure coherent policies are enroled and appropriate action is taken.

Existing assessments of cascading effects rarely consult with stakeholders within the systems under consideration. The complexity of food supply chains and the limited understanding of the operations of certain actors within these chains, suggest that direct consultation with key stakeholders may be helpful in identifying cascading effects, beyond primary production, or to validate proposed narratives^49^. Case studies are often under-utilised when developing cascading narratives and may be of particular relevance in the case of the UK, given the number of nation-wide shocks to food supply in recent years. There are some particularly pertinent examples of instances where climate change effects on food production cascaded into food security and beyond (e.g. food price spike of 2007/2008^71^). There are also many recent examples where non-climate shocks have propagated in unprecedented ways throughout the national FS. These include food shortages after Brexit^72^ and the COVID-19 pandemic^73^, as well as the observed rise in levels of malnutrition as a result of the recent cost-of-living crisis^74^. Regardless of the root cause, failings in the FS quickly surfaced as indirect effects, and it is likely that the mechanisms underpinning these effects would be similar to those associated with climate change shocks. While informative, non-climate related case studies do not allow the prediction of the lesser-known impacts of climate change on post-primary production which may have implications beyond food shortages (e.g. reduced workers productivity; increased need of cooling; disruption to critical infrastructure). Therefore, a combination of case studies and stakeholders’ consultation could be effective in producing cascading climate effect narratives relevant to FSs.

**Fig. 4:**
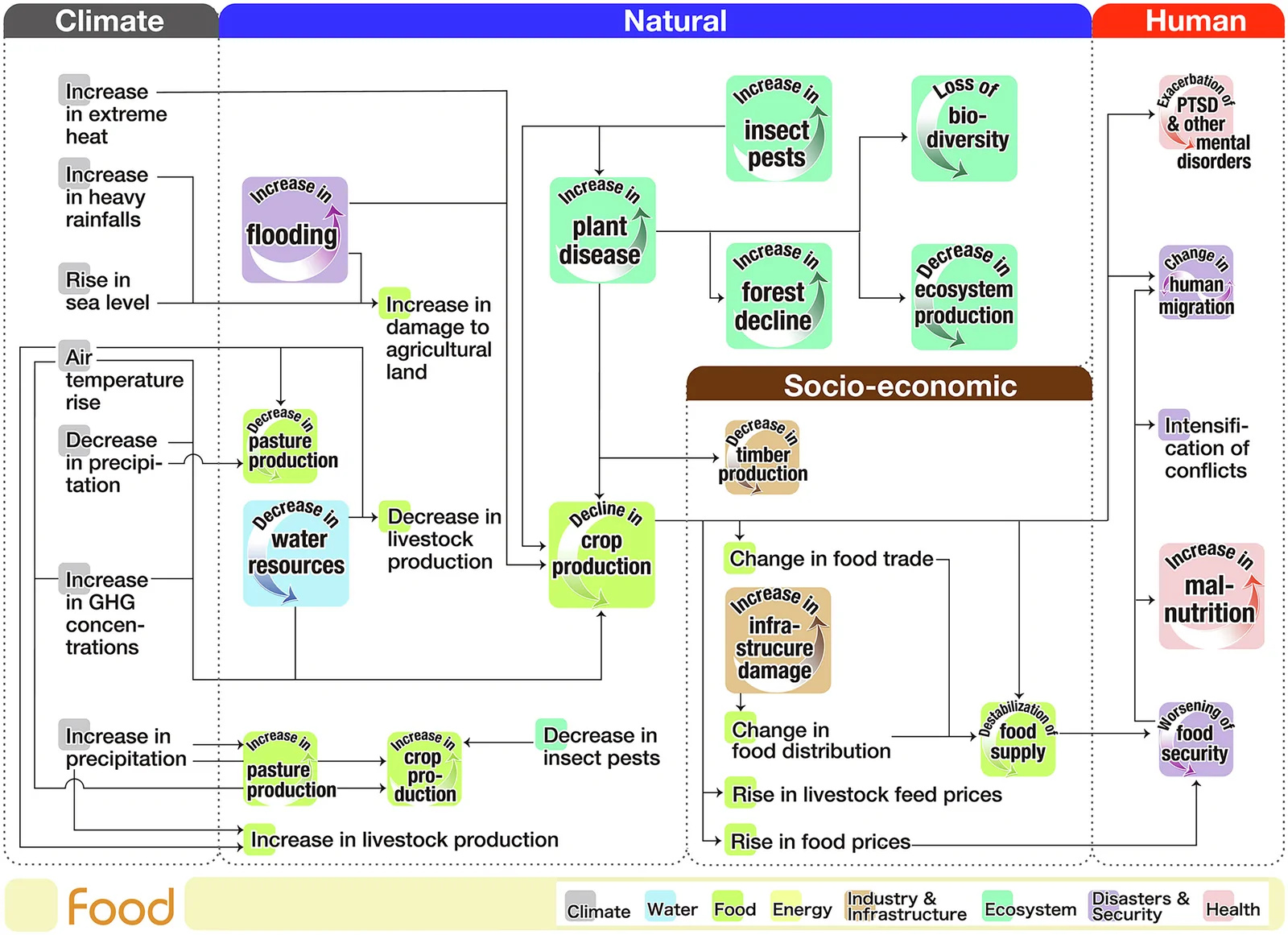
Example of the Network Flow Chart (NFC) framework. Example shows the cascading effects of climate change on food security and plant disease^63^.

Given the importance of system-level interventions for building resilience, a crucial aspect of future research is the clear conveying of complex cascading pathways to stakeholders with the power to instigate change. It is unlikely that such stakeholders will be either climate change or FS experts; therefore, the complex nature of cascading effects needs to be accurately determined and then presented in a manner that gets across the issue’s severity whilst not over-complicating the message. Effective communication is currently hindered by the lack of a standardised framework for the visualisation and analysis of cascading effects. Although numerous studies adopt a qualitative approach to describing cascading effects through in-depth and insightful discussions, these do not inherently facilitate the identification of common trends. Ultimately, any framework and its implementation face a trade-off between efficacy through detail, and readability. As many vulnerabilities in the UK FS stem from the same underlying causes, accessibility to policy makers is arguably more important than excessive detail, particularly in publications intended for wider societal engagement. One potential solution is to pair different visualisation methods, within the same study, to convey the mechanisms and implications to different audiences. For example, a CLD (Fig. 3) aimed at researchers who are familiar with the layout, and a summary of the key cascading pathways and resulting consequences in a more accessible diagram intended for policy makers and the general public. In fact, it is not uncommon for publications to develop cartoon style figures showing chains of effects, but these often omit too much detail or move away from a systems focus. An ideal pairing for a CLD could be the ‘Network Flow Chart’ concept (Fig. 4) that is based on Force Directed Graph Drawing^75^, a method for visualising complex network data that produces simple yet informative flowcharts capable of highlighting key cascading effects to any reader. Such pairing could pave the way towards a standardised visualisation framework that would facilitate effective communication and dissemination of findings.

This work highlights the need for more research on the effects of climate change on post-production stages of a FS. Bridging the gap in knowledge regarding the function and interactions of supply chain actors is crucial to identify pathways through which cascading effects could arise. The scoping review identifies a number of systemic weaknesses of the UK food system, on which specific recommendations to improve resilience are based. To start with, it is essential to tackle issues of power disparities in the food system. The results of research on the dependence on imports and the dominance of retailers indicate that producers and local players bear the shocks more than the overall system. In response, decentralising risks through investing in local and short stops supply chains and promoting SMEs and smallholders will in effect increase the adaptive capacity of the system.

Second, the literature highlights the importance of interventions on the system level. The intergovernmental governance environment and the absence of coordination across more than 16 governing institutions result in cascading vulnerabilities with the idiosyncratic resilience efforts of isolated jurisdictions seldom addressing risks to the system overall. These systemic risks can be mitigated by adopting systemic strategies that take into account cross-sector response loops, harmonise responsibilities and encouraging inter-stakeholder cooperation. Third, building resilience in production and supply chains is strongly supported by evidence of socio-economic and climate vulnerabilities. Limited access to healthy, affordable foods and sensitivity to international shocks indicate that improving domestic production, diversifying trade routes and adopting agile supply chain strategies can buffer against cascading effects from global disruptions. Investments in crop and livestock adaptation, as well as technological innovations such as IoT-enabled traceability and enhanced forecasting systems, are recommended to strengthen decision-making, reduce risk propagation and prevent food losses. The review identifies socio-economic factors and dietary changes as key nodes on risk cascading pathways. They have proven that economic limitations and excessive use of UFPs increase susceptibility, especially in low-income populations. Socio-economical and health risks can be diminished by implementing policies that change the paradigm of food security, to emphasise the accessibility of healthy diet in addition to promotion of plant-based and climate-resilient foods.

These recommendations offer potential routes to filling the literature gap of research combining cascading effects of climate change, resilience of FSs and resulting food (in)security. Increasing research attention to the topic alongside adherence to these suggestions could build up strong body of coherent research that would inform policy makers to increase FS resilience to climate change and as a result, safeguard future food security.

## Methods

### Database search and screening

This study employed a scoping review methodology guided by the Preferred Items for Systematic Reviews and Meta-Analysis Extension for Scoping Reviews (PRISMA-ScR)^76^ was used as a methodological and structural guide. Primary database searching was conducted on SCOPUS due to its favourable usability and it being the largest abstract database search engine of peer-reviewed publications^77^. Scoping reviews effectively summarise findings from research topics in which methods or presentation is heterogeneous^76^. Both topics (cascading effect assessment and the UK FS) lend themselves to a scoping review due to the wide variety in approaches used to assess and map each respectively. Additionally, scoping reviews provide overviews of broad themes and can map existing literature to aid analysis and identify areas for future research^78^. Both topic searches looked for key words and phrases in the publication title search string box; title searches were deemed most appropriate for the time-frame and scope of this work in order to ensure that selected publications had sufficient specificity. Both searches spanned the time period between 2014 and 2024.

### SR1—UK food system/food security

The first search maps the current state of the UK FS, particularly in terms of food security, This search used two title search string boxes, each encompassing the following keywords or phrases: (a) ‘UK’, (b) ‘Food System’ OR ‘Food Security’ OR ‘Food Supply Chain’. Following this, the returned publications were screened based on the following *exclusion* criteria:

- Specific focus on greenhouse gas emissions of the food system.
- Focusing on niche subsystems within the overall system (e.g. school food systems).
- Lack of explicit focus on the UK.
- UK Food system being used as a case study for an unrelated topic.

The resulting publications are summarised in Supplementary Table 2.

### SR2—Assessment of cascading climate change effects

The second search provides insights into how cascading effects move through systems, and the existing approaches taken to analyse and map these effects. Despite increasing recognition of their importance, cascading effects have received significantly less attention than direct effects in all walks of climate change research and studies focused on food security only began receiving any traction in 2018^8^. Due to this dearth of publications, the second search was not UK or FS specific and was instead focus on the methods used to assess cascading effects, alongside frameworks used to visualise, simplify and communicate the cascade pathways to other researchers, policy makers or the general public. This search used a one title search with the following key words and phrases: ‘Climate Change’ AND ‘Cascading’. The results were screened using the following *exclusion* criteria:

- Publications focused on overly niche cascading effects (unless considered potentially relevant).
- Focus on cascading effects that cause or exacerbate climate change or global warming itself.

Further to this search, additional relevant papers, found via citation search associated with cascading effects of climate change, were added given that they should have been identified through the initial search, but were omitted due to wide phrasing discrepancies used to describe cascade-type effects. The resulting publications are presented in Supplementary Table 3.

### Data charting process

Due to the complex and inherently qualitative nature of both FSs and cascading effects, quantification of content in the review papers via data charting will be limited. However, certain data items will be sought after to aid literature synthesis. Four key literature themes were identified:

- Climate change focus
- Cascading effect
- UK specific
- Food system specific

The search strategies were designed such that selected papers encompassed at least two of these. Each paper abstract was further assessed to determine the extent of research cross-over between cascading effects of climate change, FSs and the UK (Supplementary Fig. 3 presents the search strategy based on the PRISMA flowchart framework^79^).

### Collation and summary of results

The papers resulting from SR1 were further categorised, via expert assessment of the abstracts, as to whether they address the whole system or a narrower focus (i.e. diet/consumption, agriculture, local food chains, roles of social science, etc…). For each paper resulting from SR2 both the approach taken to generate cascading effect narratives and any methods/frameworks used to present these were noted. Frequency of these methods were noted to quantify existing prevalence of each method’s use in the assessment of cascading effects. Common themes in each respective review were highlighted and the frequency of their discussion in papers were charted to understand current approaches and conclusions regarding the topics researched. The results are discussed qualitatively.

### Review limitations

The scoping review used one search database (SCOPUS) and therefore only publication types available on SCOPUS were included. This offers potential for a variety of relevant sources to be missed from the search if they were not available on SCOPUS or in the forms SCOPUS allows to search for. This produces uncertainty in conclusions drawn from the scoping review related to existing literature coverage of certain topics and the proportion of their coverage. Additionally, it is acknowledged that using only SCOPUS for research evaluation could lead to bias favouring natural science and engineering over social science^80^. This is a limitation given the multi-disciplinary nature the of systemic problems discussed, wherein certain social science concepts may have been missed.

Another limitation of the scoping review is the use of title searches only. This was done in the interest of time and feasibility to ensure each paper had sufficient specificity. However, there is significant discrepancy in phrasing used to discuss cascading effects (i.e. ‘indirect’, ‘hidden’, ‘unknown’ are often used synonymous to ‘cascading’ and ‘impact’, ‘implication’ ‘risk’ ‘consequence’, ‘result’ are often used synonymous to ‘effect’) of which it was difficult to encompass all potential combinations. Any relevant publications found by citation search were added to mitigate this, but further sources may have been missed.

Regarding the analysis of scoping review results, it is acknowledged that some of the charting used arbitrarily defined variables as this was deemed to be most appropriate to chart predominantly qualitative concepts. This may present a limitation as the subsequent grouping of papers or concepts into these variables was subjective to the author. It was attempted to mitigate this through full transparency in the scoping review method section to give insight into the decision making process when charting certain data, grouping publications and developing key themes.

## Supplementary information

Supplementary Information

## Data Availability

Data sharing is not applicable to this article. The articles used to underpin the scoping review are referenced and the scoping review criteria are detailed in the methods section.
