# Supplementary material for: Assessing cascading effects of climate change on the UK food system and food security
Source: NPJ Sci Food. 2026 Jul 14;10:349. doi: 10.1038/s41538-026-00990-6 (PMC13739915; doi:10.1038/s41538-026-00990-6)
Supplement: Supplementary file 1 [file 41538_2026_990_MOESM1_ESM.pdf]

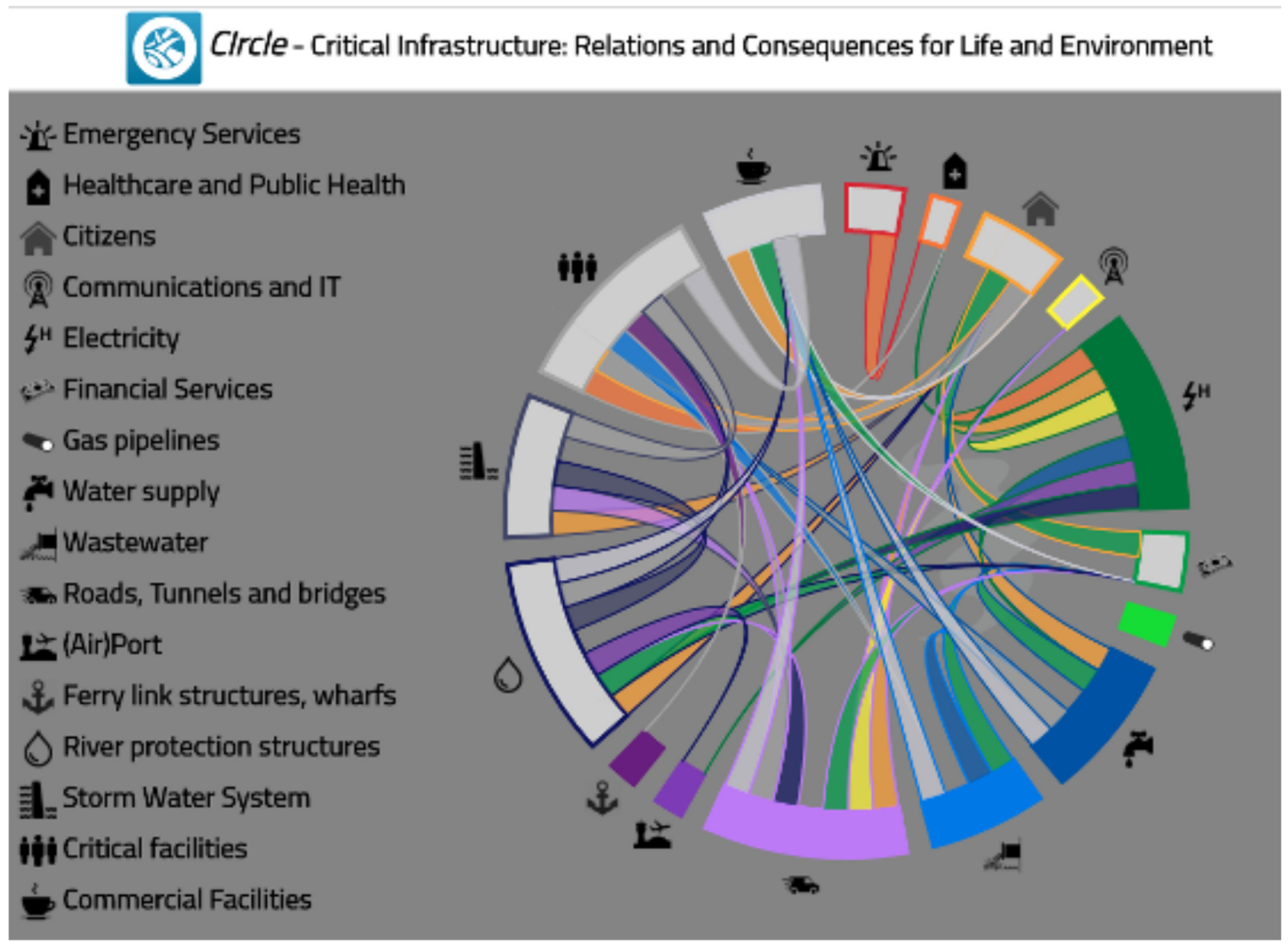

Supplementary Figure 1: Example of the CIRCLE Method.
Example represents inter-dependencies between different categories of impacts based on stakeholders' interviews.[1]

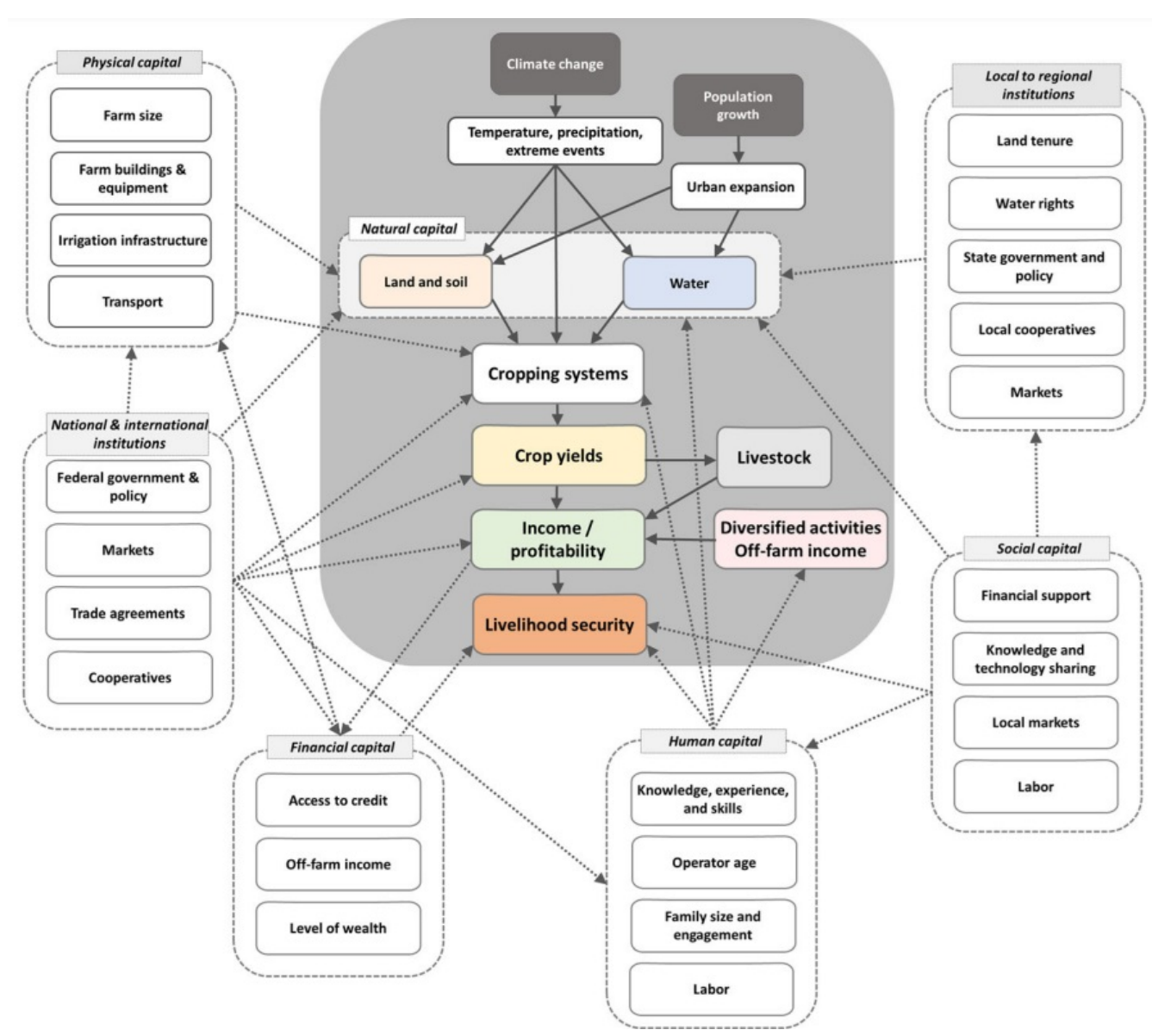

Supplementary Figure 2: Example of a system / subsystem framework.
Framework visualizes the interactions between biophysical and socio-economic factors in the context of cropland climate vulnerability.[2]

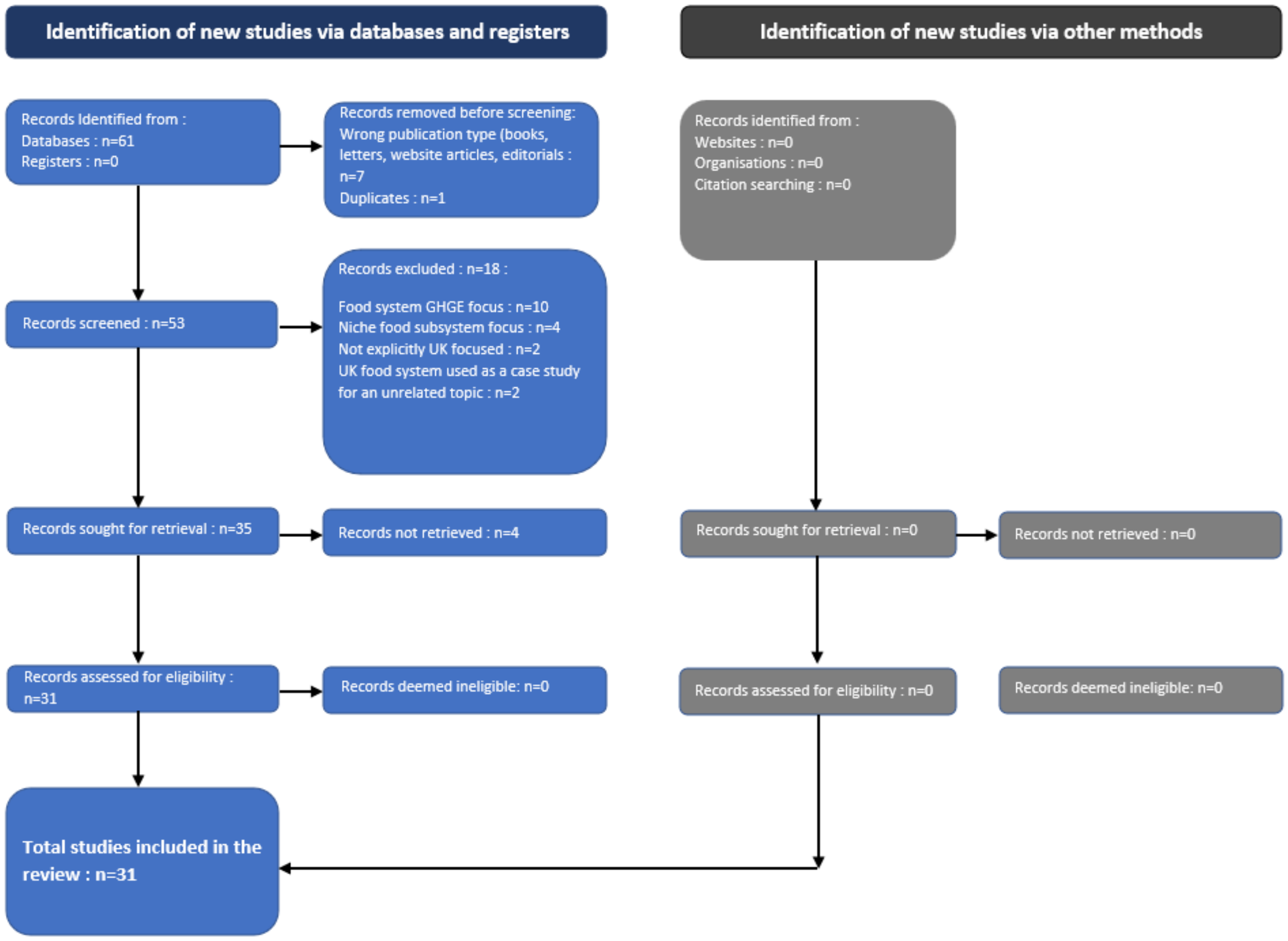

(a) Results from the UK food system publication search.

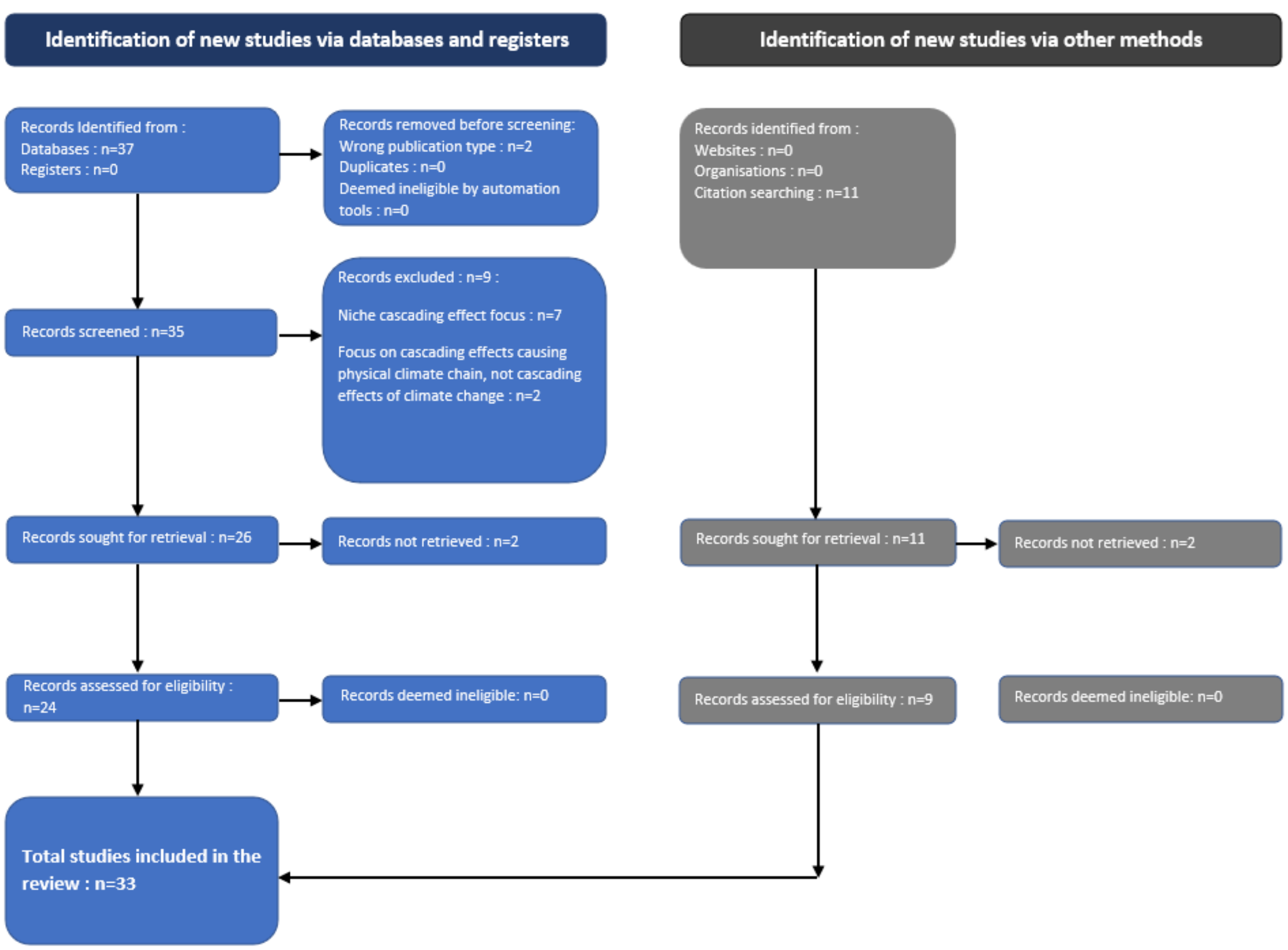

(b) Results from the cascading effect publication search.

Supplementary Figure 3: Displaying results from the respective publication searches using the PRISMA flowchart framework[3].

Hazard Area	Direct Effects on UK Agriculture	Direct Effects on UK Livestock Production	Direct Effects on UK Aquaculture
**Flooding and Sea Level Rise**	Soil run-off, soil erosion, crop loss due to over-saturation, potential infrastructure damage.	Changes to the form and structure of forage / pasture, infrastructure damage.	Coastal habitat retreat and loss, infrastructure damage, predator introduction, toxins introduction from field run-off, eutrophication.
**Extreme Events: Heat waves, Droughts and Storms**	Poor crop and grass growth, reduced yields, inability to supply winter animal feed, crop destruction in storms, reduced water access.	Animal welfare problems, reduced yield and fertility, mortality in severe cases, inadequate water access for livestock.	Fish escape, cultivation infrastructure destruction, ecological stress facilitating disease, loss of production.
**Mean Temperature Increase**	Increased pest and disease prevalence, reduced yields, reduced biodiversity.	Increased water demand for consumption, decrease in nutrient availability, decrease to animal productivity/feed conversion, worsened livestock health, decreased reproductive efficiency, reduced biodiversity.	Effected toxin, nutrient and pollutant transport facilitating disease, algal bloom.
**Increased Climate Variability**	Increased pest and disease prevalence, reduced yields, increased vulnerability to mass-produced crop clones.	Reduced productivity due to varying conditions not facilitating any given optimal farming approach, dilemma of breeding for efficiency or resilience to climate variation.	Reduced productivity due to sub-optimal farming conditions, increased vulnerability due to farming species with a narrow thermal range, changes in reproductive cycles.

Supplementary Table 1: (non-exhaustive) Summary of established direct effects of climate change on agriculture([4], [5], [6], [7]), livestock production ([8], [9], [10], [11]) and aquaculture ([12], [13], [14]) considering the UK priority risks identified from UK climate change projections ([15][16]) and risk assessment ([17]).

**Authors & Year**	**Title**	**Relevant Findings**
**1)** Black et al. (2024)	What constitutes food system resilience? The importance of divergent framings between UK mainstream and local food system actors.	Examines how key actors in UK FS understand the role of local food sectors in resilience. Actors within alternative food networks perceive the UK FS as not resilient and think local FS are a route towards greater resilience (local FS viewed within the systemic framework). Actors in the mainstream FS perceive the existing UK FS as resilient and local FS have only specific functions in the existing framework (local FS viewed as add-ons to the systemic framework or just-in-time supply chains).<br>The two groups agree that dynamic public procurement and local abattoir provision is important, but this does not change the underlying different understandings on the roles local FS play in systemic resilience. Local food actors have little say in overall food system operation.
**2)** Bhunnoo & Poppy (2020)	A national approach for transformation of the UK food system.	Describes a program for transformation of the UK food system to promote healthy people and a healthy environment. UK food supply chain currently maximize calories per hectare, whereas paper suggests that for food security, it needs to maximising number of people fed healthily and sustainably per hectare.<br>UK FS intrinsically linked to global supply chains as around 50% of food imported.<br>Climate change induced food security risk exacerbates existing problems regarding health and food system sustainability.<br>The complexity of the UK FS, with its many stakeholders, governing bodies and regulatory agencies each, with multiple and often disparaging objectives, makes coherent policy making simultaneously vital yet challenging.
**3)**Pool & Dooris (2022)	Prevalence of food security in the UK measured by the Food Insecurity Experience Scale.	Contributes to the (historically inconsistent) measuring of UK food insecurity. Reports and analyses national survey data conducted in line with UN FAO insecurity experience scale. Logistic regression used to model relation between insecurity and geographic and socio-demographic variables. 3% of sample recorded severe food insecurity, a 66.67% increase relative to most recent directly comparable UK analysis. Some degree of food insecurity reported by 14.2% of sample, typically higher among younger ages, lower incomes and home renters (as opposed to owners).<br>No significant geographic pattern to the distribution of food insecurity.
**4)** Tsolakis & Srai (2017)	A System Dynamics approach to food security through smallholder farming in the UK.	Investigates the role of smallholder farming in increasing food security and sustainability. Discusses relevant literature and develops a system dynamics modelling framework to capture self-sufficiency of cereals produced in the UK. Provides an initial approach to developing food security early warnings systems and decision support tools to be employed by policy makers. Appropriate governance and effective policy making (that supports smallholding / short supply chains) promise to be useful in ensuring food security and social cohesion while not compromising economic growth and sustainability. Lack of systemic approaches to capture dynamic nature of food security phenomenon. Thus system dynamics approach should be considered when investigating food security in developed countries due to the multidimensional and complex nature of the issue.<br>Policy increasing smallholder prevalence in cereal production could prolong food security, increase average gross domestic profit, improve cereal productivity related to fertilizer use and have positive employment and public health implications.
**5)** Hunt et al. (2024)	A critical exploration of the diets of UK disadvantaged communities to inform food systems transformation.	Performs a scoping review of UK peer reviewed qualitative literature regarding the diets of disadvantaged communities to inform food system transformation. Aims to determine level of existing data available to explore the diets of the disadvantaged and to what extent literature links individual experience to wider food system aspects. Research into the diets of disadvantaged communities provide varied findings which commonly focus on disadvantage at an individual level. Links to supply chains and food systems are limited.<br>Systems thinking required to tackle the issue of poor diet among disadvantaged as a food system, not individual, issue.<br>Future research needs to prioritise more nuanced themes such as socio-economic interactions, affordability and access issues while adopting knowledge exchange, method uniformity / co-production in research to allow clearer synthesis of research to inform food system changes.

Supplementary Table 2: Summary of Articles Included in Scoping Review 1 (Part 1).

**Authors & Year**	**Title**	**Relevant Findings**
**6)** Alae-Carew et al. (2022)	The role of plant-based alternative foods in sustainable and healthy food systems: Consumption trends in the UK.	Examines plant-based alternative foods (PBAFs) consumption trends in UK via cross-sectional food consumption data, and assess food system implications. Proportion of individuals reporting consumption of PBAFs increased from 6.7% (2008) to 13.%(2019). Females more likely than males to increase consumption, millennials generation most likely to report PBAF consumption, low meat consumers more likely to consume than high meat consumers.<br>Results support the hypothesis of the important role of PBAFs in the transition towards sustainable food systems in the UK by demonstrating their increasing popularity.
**7)** Attorp & Hubbard (2022)	Governing the UK agri-food system post-Brexit.	Considers the challenges facing the UK food system regarding sustainable practice post-Brexit. Aims to shed light on the ways different actors in the UK FS interact with the debate on which aspects of sustainability should be prioritised and shape policy as a result. Power in the UK FS lies with retailers, processors and consumers. UK FS governed by many bodies (16+) and DEFRA relies on over 30 agencies/public bodies for its remits, in which responsibilities of each are not well defined. This causes policy disconnect which hampers policymakers' ability to tackle complex systematic problems.<br>UK FS relies on just-in-time supply. This benefits efficiency and inventory but renders the system more vulnerable to disturbances upstream (which are increasingly likely as climate change makes global food supply chains more volatile). The economic benefits of just-in-time supply are unevenly distributed in favour of retailers and processors. Addressing equal policy focus to each aspect of sustainable food systems (food security, social sustainability and environmental sustainability) requires power imbalances in the food system to be redressed.
**8)** Brewer et al. (2019)	The Internet of Food Things: Adding value to the digitisation of the UK food supply chain.	Provides detail on the 'Internet of Things (IoT) Plus' and investigate how it can be integrated into FS digitisation. The IoT is having increased impact on the digitisation of food manufacturing chains and it will be a key component in managing the increasing complexity of food systems.<br>Many stakeholders in the food manufacturing chain are enthusiastic about a digitised food network via the application of IoT. Authors believe complex technological solutions will be intrinsic to the future of the food supply chain and that these possible solutions will enable new business models that go beyond finance to embody social and community dynamics.
**9)** Cole & Petrikova (2024)	UK and Global Food Security in the Era Of 'Permacrisis'.	Analyses the UK's national and global food security in the permacrisis era from the perspective of global geopolitics. Regarding food security, the focus is on the macro-level pillars: food availability and food stability. The UK is heavily import reliant, therefore UK food security is connected to the global food system. The latter is becoming increasingly volatile due to climate change and conflict impact on production and politics. Cascading effects will make the UK increasingly vulnerable. UK food security could be enhanced by diversifying food and other trade routes and increasing domestic production via technological innovation (in which vertical farming is emphasised). To achieve the latter, subsidies and support policy encouraging agricultural production and innovation could be built into future resilience and food security plans.
**10)** Davie et al. (2022)	UK heatwave impacts on agrifood: implications for a climate-resilient food system.	Explores the impacts of heatwaves and record temperature levels using UK poultry and wheat sectors. Discusses adaptation requirements for climate resilience alongside a net-zero UK food system and consider future needs. Impact of extreme heat on primary production: livestock loss due to heat stress most prominent, wheat yield very dependent on timing of heat stress within a crop cycle. The greatest effect on retail is the increased cold chain requirements and associated costs.<br>There is a trade-off between heat resistance adaptation measures and yield/quality, more long term economic assessments will be required to optimise these two simultaneously.<br>Data advances and seasonal forecasting will likely be important to aid decision making of primary producers.<br>A major challenge is determining whose responsibility it is to action and fund adaptation strategies within the food supply chain, making sure burdens are spread equally.

Supplementary Table 2: *Continued* (Part 2).

**Authors & Year**	**Title**	**Relevant Findings**
**11)** Do et al. (2021)	Supply chain agility responding to unprecedented changes: empirical evidence from the UK food supply chain during COVID-19 crisis.	Investigates how supply chain agility practices were implemented during the COVID-19 pandemic and presents expectations on how these actions could transform the supply chain in future crises. Following the COVID-19 pandemic each affected element of the food supply chain pursued agile responses through sensing (anticipating/ identifying /assessing threats) and seizing (utilising available resources) capabilities. Permanent supply chain transformation requires firms and their supply chain to develop a sustaining capacity to ensure the measures taken during the crisis outlast the crisis. The UK FS's relatively swift and effective response to the COVID-19 pandemic highlights the potential benefits of supply chain agility practices as a mitigation strategy to safeguard food security in light of climate shocks. The agility concept is more redundant regarding slow onset effects of climate change.
**12)** Edmondson et al. (2019)	Grow your own food security? Integrating science and citizen science to estimate the contribution of own growing to UK food production.	Estimates current levels of UK own-growth fresh fruit and vegetables (FFV) and how this could be increased if more urban land was made available for own-growing. Aims to provide evidence based support to the assumption that own-grown FFV production can increase food security. Urban areas are heavily reliant on traditional agriculture, which faces yield issues as a result of continued soil degrading practices alongside climate change.<br>Commercial agriculture heavily relies on a seasonal migrant workforce due to UK citizens' unwillingness or inability to do physical farm work. The migrant workforce may be less available due to Brexit.
**13)** Elliott & Bhunnoo (2021)	Scenarios for transforming the UK food system to meet global agreements.	Explores four future food system scenarios for the UK and assesses implications on dietary shift, food waste, biodiversity and food price for each scenario. Scenarios and resilience resulting from a change to a food system can be very different depending on which UN Sustainable Development Goals (SDGs) the transformation prioritises. Any change is likely to induce food price increases, but the civil unrest caused by price rises may be more significant when the transformation is driven by climate mitigation over holistic sustainability. Land-use is directly related to biodiversity and this highlights the need reduce land intensification and footprint.
**14)** Garnet et al. (2023)	Future foods: Morphological scenarios to explore changes in the UK food system with implications for food safety across the food chain.	Examines systemic change in food systems to inform policy makers in their management of uncertainty and shaping of food policy. A number of alternative food system scenarios are presented (developed in collaboration with the Food Standards Agency). Transformation of the food system in the UK is dependent on the direction the global food system takes: either towards embracing global trading or entering resource tension. The former provides trend of positive changes to production and consumption habits wherein consumers embrace a more responsible diet. Entering a state of resource tension has the opposite effect in which a declining food economy and increased food prices reduce diet quality to more processed and less nutritional foods, although these may be more resilient to emerging food safety issues. Food system development is dependent on both technological and socio-economic developments that dictate vulnerabilities in food supply chains as well as the adaptive capacity of different actors.
**15)** Jin & Zhu (2023)	Facing up to the UK's Food Crisis—A Design Exploration of the Self-Sufficient City for Resilience and Food Security.	Reviews the potential of future food shortage in the UK and investigates design strategies for creating a more sustainable agrarian urbanism, looking at three case studies of urban utopias. Food supply system needs to be resilient to natural phenomena (climate variability, extreme weather) as well as socio-economic and geopolitical *upheavals.*<br>Existing food supply crisis in UK exacerbated by Brexit, and relying on former colonies outside of EU for food supply is unethical, hence paper suggests self-sufficiency is needed. Rural city development may offer enhanced self-sufficiency: a "decentralised rural metropolis with cutting edge farming systems" could offer a solution in which an expanding population can respond to future food supply uncertainty (climate induced or otherwise).

Supplementary Table 2: *Continued* (Part 3).

**Authors & Year**	**Title**	**Relevant Findings**
**16)** Johnstone & Lonnie (2024)	Tackling diet inequalities in the UK food system: Is food insecurity driving the obesity epidemic?	Explores evidence for solutions in the retail food environment to support positive change to the UK food system in light of food inequality, insecurity and the obesity epidemic. Approaches focusing solely on individuals' responsibility and consumer education are insufficient to improve population health: a whole system approach is needed with interventions that will be complementary and synergistic.<br>Indicators of good health are not spread equally or fairly across the UK population. Policy intervention needs to address the unequal availability of healthy food options. The authors advocate for a co-creation approach to research into food system transformation that give value and power to the lived experience of people experiencing food insecurity.
**17)** Hasnain et al. (20201)	Mapping the UK food system–a report for the UKRI transforming UK food systems programme.	Quantifies the distribution of economic value, number of enterprises and level of employment across the UK food system. Provides the first assessment of the overall shape of the UK food system. Concentration in the UK economy has increased with time: small number of large retailers dominate the food supply chain, two major players dominate contract catering and a few US entities dominate fast food.<br>30% of food manufacturing sector employees come from the EU. Employment in the food sector is characterised by low-wages and a lack of appropriate skills. UK has the third highest volume of sales in ultra-processed food (UPF)/capita out of 80 high and middle income countries, and the most processed diets in Europe. It is facing an obesity epidemic. UK heavily import reliant, even for foods that can be grown domestically. UK has a trade deficit in all food categories except for drink (due to high levels of UK whiskey exports).<br>Data gaps exist in: enterprise breakdown in food retail, catering and wholesale, the proportion of food related packaging and the proportion of food-related employment, enterprise and turnover in distributing/storage activities.
**18)** Lee (2015)	Educating for food security in the UK: Planning for an uncertain future.	Investigates the context of higher education delivery for the next generation of agriculture and commercial horticulture graduates. Reviews the impact of resource restrictions and opportunities of novel teaching methods. UK agriculture and commercial horticulture that can secure enough food reliably for all citizens should be given priority, but this will require radical changes to food production to produce good yields and be resilient to a changing climate. There is an urgent need to plan for university degrees in agriculture/commercial horticulture which can be delivered as 'cloud based' courses. Author perceives this as important and necessary for the development of a new generation of agriculture graduates equipped to ensure future food security through domestic production, though this will require institutional adoption and implementation.
**19)** Mattevi & Jones (2016)	Traceability in the food supply chain: Awareness and attitudes of UK small and medium-sized enterprises.	Examines awareness and attitudes towards traceability in the food supply chain within UK small and medium-sized enterprises (SMEs). Tests propositions in the literature to understand whether SMEs are aware of the main objectives of traceability, what importance this is given and their intension to invest. SMEs are aware of the main purposes of traceability systems, i.e: cost reduction, rapid recall and improvements to food safety and quality. But there is a misguided belief that traceability can reduce probability of recalls. General attitude towards traceability is positive in terms of recognition but there is lack of willingness to invest in traceability system enhancement.

Supplementary Table 2: *Continued* (Part 4).

**Authors & Year**	**Title**	**Relevant Findings**
**20)** Mattevi & Jones (2016)	Food supply chain: Are UK SMEs aware of concept, drivers, benefits and barriers, and frameworks of traceability?	Investigates the drivers for the implementation of a traceability system among UK SMEs in the food supply chain. Fills the gap in the literature to investigate concepts, drivers, benefits, barriers and frameworks of traceability of UK SMEs operating in the food supply chain. Main drivers for increasing traceability: safety and quality, even more so than regulation. These are strong external drivers. Technology largely not seen as a driver to implement traceability systems<br>Lack of implementation seen in general, but especially in small firms who might need external help in enhancing their systems. Supply chain traceability not widely implemented, usually companies trace material during the production process. Lack of regulatory compliant respondents suggests governments and associations should push SMEs' education on regulatory requirement.<br>Benefits of traceability (continuous monitoring, more rapid recall, compliance, safety, quality, supply chain resilience), outweigh barriers (cost, difficulty accessing suppliers information, information limitations, lack of skilled staff and lack of standardization / transparency). Despite this, many benefits appear unknown to SMEs (supply chain efficiency, logistics/inventory management gains, product differentiation, brand name protection, competence development and best practice documentation).
**21)** Milbourne & Coulson (2021)	Migrant labour in the UK's post-Brexit agri-food system: Ambiguities, contradictions and precarities.	Explores the role of migrant labour within the UK agri-food system and how the roles of migrant workers are perceived by different food supply chain actors regarding farming, food and Brexit. Dominant actors in the UK food supply chain have used Brexit to construct a crisis of migrant labour supply, arguing for new policy mechanisms to guarantee the future provision of low-cost imported labour.<br>Presence of migrant labour has been normalised and institutionalised by conventional food organisations, but the realities of migrant work and voices of migrants themselves has been made clear in their absence: horticulture in the UK just as dependent on migrant workers as it is on climatic conditions and soil type.<br>This approach shows reluctance to engage with the broader agenda for change which was provided by the complete shock to the sector during Brexit: the opportunity to develop a new policy framework, address sustainability and resilience in light of Brexit was largely shunned by actors in favour of protecting cheap labour supply.
**22)** Morris et al. (2019)	Openness to Social Science Knowledge? The Politics of Disciplinary Collaboration within the Field of UK Food Security Research.	Explores the 'knowledge politics' played out between universities and research institutions as sites of expertise in the agro-food domain. Investigates the openness of the domain to the expertise of agro-food social sciences, particularly the requirement of interdisciplinary collaboration and how the research field's diversity is understood by food system actors. Paper argues that food security research in the UK is only open to certain social science knowledge and this is likely to negatively affect the ability for addressing the challenges of food security.<br>The importance of social science in tackling food security is generally appreciated and the research field is open in principle, but evidence suggests research and action is dominated by natural science.<br>To facilitate collaborative working between disciplines in agro-food research fields, agro-food knowledge, politics scholarship and the governance of knowledge making needs to change.
**23)** O'Keefe et al. (2016)	Consumer responses to a future UK food system.	Explores consumer responses to potential change in food-related practices to mitigate and adapt to climate change via focus group workshops. Changes that could be linked to existing competencies (cooking and preparing) viewed more positively, lack of competency appeared a key barrier to accommodating change.<br>As of time of study, climate change and sustainability did not influence food purchase decisions. Financial pressures much more prevalent in the conversation. Policies encompassing multiple benefits (in which climate is one, but there are other more pressing incentives for the consumer) argued to have more benefit / potential; So-called "win-win" policies.

Supplementary Table 2: *Continued* (Part 5).

**Authors & Year**	**Title**	**Relevant Findings**
**24)** Poo et al. (2024)	Global Food Supply Chain Resilience Assessment: A case in the United Kingdom.	Develops a new method to assess food supply chain resilience at a country level by combining the two most relevant (subjectively) attributes: a country's production to supply ratio and shipping transport connectivity. Logistics and connectivity to global food networks underpin food security and highlights the need for integrated approaches to resilience.<br>The papers devised method of generating a National Food supply chain Resilience (NFR) index shown to provide an adaptable quantitative method to assess FSC resilience across a broad spectrum of conditions. Quantitative thresholds can act as early warning signals to allow governing bodies to anticipate and manage disruptions Local food production and transport connectivity identified as key factors to national resilience while the observation of different indices for different food types shows the need for customised approaches to achieve resilience. Adaptive strategies and continuous monitoring will be required to maintain food security. Solutions should consider the interaction of local production, global trade networks and socio-economic factors and thus take a systems approach to shape resilient food systems.
**25)** Rothwell et al. (2022)	A new direction for tackling phosphorus inefficiency in the UK food system.	Presents results of a detailed phosphorus flow analysis regarding the UK food system in 2018 and subsequently highlighting priority areas for systems intervention to improve sustainability and resilience of phosphorus use in the UK food system. UK relies heavily on phosphors imports. Three key inefficiency hotspots were identified: agricultural soil surplus and accumulation, loss to aquatic environments and waste disposal to landfill and construction (almost entirely from incineration ashes from food waste).<br>New strategies are required to promote recovery from manures, bio-solids and food system waste to reduce phosphorus accumulation and loss, thus reducing import reliance without compromising food systems reliant on phosphorus. Substance flow analysis represents a novel method which offers potential to inform these strategies by identifying hot-spots of inefficiency from local to national scales.
**26)** Shaw et al. (2024)	What does 'co-production' look like for food system transformation?	Identifies and maps examples of co-production methods employed across the Transforming UK Food Systems program and identify key principles for co-production. Co-production approaches are increasingly prevalent in food system transformation strategies but there is a lack of consensus as to what this should look like. The 'messiness' of co-production must be embraced and accepted by researchers and it is important to take a systems approach to co-production and acknowledge the dynamics of the food system, diversity of perspectives and non-linearity of research. Paper findings reinforce that co-production needs to be principle driven, there is no single method or approach. Four key principles of co-production identified: Relationships Knowledge, Power and Inclusivity.
**27)** Shittu et al. (2022)	Investigating how COVID-19 has challenged the Eurocentric concept of 'development'.	Investigates how the COVID-19 pandemic challenged the UK's development status by analysing how the pandemic has impacted the country's food industry. Paper argues that the ideal future UK food system would consist of a combination of short and longer food supply chains and a more balanced involvement of urban and rural agriculturalists in production processes.<br>There is a clear power imbalance in the food system: system is retailer-controlled and producers have to rely on middle men to communicate with these retailers. The feelings and roles of these middle men are hard to identify as their existence relies on the power imbalance, hence any opinions offered on the state/true operation of the system are likely to be biased.<br>Euro-centrism is deeply ingrained in British culture and decoupling from this is likely to be lengthy but necessary for systemic benefit, including benefit to the food system.

Supplementary Table 2: *Continued* (Part 6).

Authors & Year	Title	Relevant Findings
**28)** Tak et al. (2024)	Who is responsible for fixing the food system? A framing analysis of media reactions to the UK's National Food Strategy.	Identifies frames used to shape food system policy intervention to fix the UK food system deemed broken due to high levels of child obesity, stunting and a lack of coherent food policy. Develops a framing analysis on traditional media and sentiment analysis of twitter reactions to the National Food Strategy (NFS). Media coverage of the NFS used cultural wars to shape the debate of who is responsible to fix the food system (government vs industry vs people).<br>Media framing (negative scaremongering and criticism of state intervention to solve structural causes of food system inequalities) facilitated the government publishing a delayed, weak and non-committal response to the recommendations. Industry believed to be equipped to intervene on its own accord as the findings in this paper reiterate the narrative in which governments prefer to emphasise individual responsibility rather than systemic interventions, as is emphasised by right-leaning media.<br>NFS comments on diet recommendation were received as attacks on the poor, that they over suggested helplessness and lack of control. However voices of the poor were omitted by traditional media. Media's depiction of culture wars is unproductive, instead they should promote discussion of intervention which reform the systemic causes of the 'UK's broken food system'.
**29)** Vicario et al (2023)	Synergies and trade-offs in drought resilience within a multi-level UK food supply chain.	Analyses the synergies and trade-offs in drought resilience in a multi-level food supply chain, using the UK potato supply chain as a case study. Results suggest the potato supply chain increased its resilience to weather extremes through retailers and packagers having a wider spread of supply, an increasing resilience on forward contracts and favouring growers with established water security.<br>Resilience building measures taken by supply chain actors are designed to reduce their own risk and hence do not consider other parts of the chain or the entire system, which may lead to overall failure in achieving the desired resilience outcome, or place strain on other actors (typically producers). Highlights the need for a more integrated, systems approach in order to build trust and cooperation between actors.<br>UK FSC resilience actions dominated by short-medium term actions with little contribution to future resilience: financial pressures and uncertainty regarding regulation and politics force producers to focus on short-term efficiency, holding back progress towards longer term resilience.
**30)** Yawson et al. (2020)	Exploring the impacts of climate change and mitigation policies on UK feed barley supply and implications for national and transnational food security.	Assesses the impact of UK climate mitigation policies on future food penalties (using feed barley and meat) to highlight potential implications for transnational food security. Land area under climate mitigation scenarios may considerably reduce the land available for barley, and cause resulting deficits in domestic feed and hence meat produce.<br>The UK would have to move away from net exporters to significant net importers of meat and barley. These increased imports would require adjustments in trade flows to other top importers from these countries.<br>The magnitude of projected deficits highlight the adverse impacts of UK land-based climate mitigation on its food security. Effects could cause food security issues in developing economies that rely on the global market for feed barley and meat as their market shares would have to reduce in order for the UK to occupy significantly more.<br>Risk of tele-coupled impacts of mitigation measures have yet to be factored into climate adaption, especially in developing countries. Effects on socio-economic and political circumstances that allow access to food on the global market are broadly unknown.
**31)** Yawson et al. (2017)	Effect of climate and agricultural land use changes on UK feed barley production and food security to the 2050s.	Assesses the effects of projected climate and land use changes on barley feed production and, consequently on meat supply from 2030 to 2050. While UK spring barley production will remain viable and potentially thrive in changing climatic conditions, total land area allocated to barley production ultimately determines UK self-sufficiency.<br>Land area expansion and/or significant yield increases are required to prevent large deficits in domestic production of livestock feed. This will cascade to future meat supply and potentially exacerbate UK's import reliance.<br>Food security policy needs to consider the effect of agricultural land changes on key crops such as barley: theoretically imports can make up for this increasing deficit but where these will come from and whether the UK should increase import reliance are pertinent questions.

Supplementary Table 2: *Continued* (Part 7).

Authors & Year	Title	Relevant Findings
**32)** Cradock-Henry et al. (2020)	Elaborating a systems methodology for cascading climate change impacts and implications.	Expands upon system-based methodology to identify and evaluate cascading risks of climate change. Application of this given using participatory process with urban infrastructure facing effects of damaging earthquakes and the prospect of future climate change. The interconnectedness of domains causing circular and potentially repeating cascades can be visualized effectively via a system dynamics causal loop diagram (CLD).<br>CLD final step of a 5 step procedure entailing initial problem definition, mapping of critical infrastructure, generating narratives of cascading impacts, using these and modelling software (e.g. Vensim[18]) to develop the CLD, which can then be evaluated and refined.<br>Application of this method to wider systems (e.g. food) may add knowledge of particular domains.<br>Future research could zoom into specific nodes to validate the narratives shown in a system CLD.
**33)** Lawrence et al. (2020)	Cascading climate change impacts and implications.	Discusses the origins of cascading impacts thinking, presents results of an investigation into cascading impacts and implications in New Zealand. Investigation: participatory and collaborative approach taken through workshops and semi-structured interviews with sector informants. Consideration of combined effects of linked stressors facilitates a better understanding of the scope and scale of climate change impacts.<br>Fewer paper analysing cascades from slow emerging and ongoing climate change impacts.<br>Smaller cycles in a causal loop diagram can be isolated to facilitate step-wise exploration and discussion on specific cases.<br>Linear consideration of impacts can conceal the feedback loops effects propagate in, leading to sub-par responses or policy.
**34)** McIntyre-Mills (2018)	Cascading risks of climate change on water security and the potential for rapid adaptation Consequences of modernity', potential of the double hermeneutic and implications for human security.	Explores the cascading interconnected challenges associated with energy and water shortages. Focuses on the impact of urbanisation in the context of climate change, specifically in Cape Town. Rapid adaptation to conserve water in Cape Town was achieved through a transparent water mapping system and donations from farmers and NGOs elsewhere in South Africa.<br>Fear for the future and desire not to be shamed were drivers for instigating fast adaptation.<br>Case made that cascading effects of climate change (in the Western Cape region of South Africa) can be addressed by redressing rural-urban imbalance in development opportunities.<br>Multiple holistic pathways to better understand and mitigate cascading effects offered in concluding remarks.
**35)** Semenza (2020)	Cascading risks of waterborne diseases from climate change.	Reviews the effects climate change can have on waterborne disease. Climate induced disruptions to the hydrological cycle via rainfall changes, droughts and temperature rises can initiate an effect cascade that increases prevalence of waterborne diseases through a variety of different pathways.<br>Active surveillance and early warning systems offer potential interception and early mitigation strategies (ECDC Vibrio Map Viewer[19] and One Health Surveillance Strategy[20] discussed).<br>Supply side investment/policy intervention likely required to actuate these systems.
**36)** Groundstroem & Juhola (2021)	Using systems thinking and causal loop diagrams to identify cascading climate change impacts on bioenergy supply systems.	Utilises systems thinking to develop an analytical framework to address the gap of research regarding cascading effects of climate change on the bioenergy supply chain.<br>Greater number of connections in a CLD encompassing climate effects means potential for cascading effects to be wider reaching.<br>Higher importance cascading impacts can be identified from a CLD, alongside feedback loops. The greater the tendency of feedback loops to be re-enforcing, the more likely it is that the system will be highly dynamic in future change.<br>No framework or model can capture all causal links: including all potential subsystems related to the system of interest will create an abundance of loops and causal links; any causal loop diagram inevitably serves as a simplification of the supply network and related vulnerabilities. Regardless, models are vital in beginning to understand inter-connections in a system to prevent ill-fitting policies.

Supplementary Table 3: Summary of Articles Included in Scoping Review 2 (Part 1).

Authors & Year	Title	Relevant Findings
**37)** Ciapessoni et al. (2023)	A Cost–Benefit Analysis Framework for Power System Resilience Enhancement Based on Optimization via Simulation Considering Climate Changes and Cascading Outages.	Proposes a cost-benefit analysis framework based on optimisation via simulation selection of an optimal range of protocols to enhance resilience. Identify risk indicators for the cost-benefit analysis, accounting for likely models of climate change. Cascading risks/outages accounted for. An optimal approach to increasing resilience via relevant passive and active measures could be found via an Optimisation via Simulation and scenario-based methodology that has the ability to account for climate change effects and potential cascading effects. Such frameworks are flexible and can have wide application.<br>Findings included that the developed framework aided decision making in prioritising interventions to the problem studied (power system resilience), and it is suggested that the framework be applied to other natural threats to aid resilience enhancement.
**38)** Steele et al. (2018)	Cascading impacts of climate change on southwestern US cropland agriculture.	Assesses the vulnerability of cropland agriculture to challenges that result from climate and non-climatic factors. Aims to demonstrate how in different location, different cropping systems exposed to similar change in climate may experience different changes in vulnerability from various contextual factors. Framework for mapping the scope of considerations visualised. Traditional biophysical vulnerability assessments provide data on potential impacts on specific primary production aspects and can guide technological solutions, but vulnerability needs to also be assessed contextually, and not linearly, to facilitate adaptive solution generation.<br>In extreme conditions, existing technological advances that help farmers cope with climate change may push cropland beyond the ability to cope.<br>Contextual vulnerability consideration allows determination of practical financial, institutional and policy level responses/resources that will be necessary to help farmers adjust to new conditions.
**39)** Teshome (2024)	Charting the systemic and cascading impacts of climate change on marine food systems and human health.	Provides a note on the systemic and cascading impacts of climate change on marine food systems and implications for human health. The ocean is key to the production of nutrient-rich marine foods, climate change induced shifts in production of fish species poses a threat to food security.<br>Global food system encompasses large socio-economic disparity; promoting food sovereignty and harnessing indigenous knowledge to reframe the rights of local resource users could be of benefit. As such, local food systems support through local fishery management will be increasingly important.<br>Effective contribution of seafood to the global food supply will depend on fast, effective and continuous mitigation and adaption action encompassing ecological, economic, policy and technological influence.<br>Seafood under-researched in recent literature on food security.<br>Short–medium term adaptation should focus on equity and support of producers and local consumers reliant seafood.
**40)** Bengston et al. (2022)	Accelerating Climate Change: An Exploration of Cascading Future Implications.	Explores the cascading effects of accelerating climate change, using the Implications Wheel method to identify and score more prominent higher-order effects of accelerating climate change. Analysis of many 2nd/3rd order implications generated. Structured implication brainstorming generating exponentially more impacts at higher order implications (I.e. 3 1st order, 29 2nd order, 113 3rd order), atypical of less structured thought processes.<br>The Implications Wheel approach favours the recognition that positive innovation comes from sheer numbers of generated ideas.<br>There is a growing need for better foresight into the dynamics of cascade effects from climate change to guide policy.
**41)** Huang et al. (2020)	Assessment of the economic cascading effect on future climate change in China: Evidence from agricultural direct damage.	Assesses the economic impact of climate change in China. Shifts the focus of agricultural climate change research from direct effects to cascading effects into other sectors, particularly the economy. Observations from China case study: significant amplification effect caused by direct agricultural economic damage as this cascades throughout systems in the nation.<br>Agricultural damage and economic losses has notable but different effects on different systems/sectors (manufacturing sector most notable).<br>Decrease in cultivated land area has significant effect on agricultural sector and economy via cascades. Dual effects of temperature increase and loss of cultivated land amplify economic cascade effects.

Supplementary Table 3: *Continued* (Part 2).

Authors & Year	Title	Relevant Findings
**42)** Zhou et al. (2023)	A bibliometric review of climate change cascading effects: past focus and future prospects.	Provides a systematic overview of existing literature regarding the cascading effects of climate change. Scientometric analysis of literature dated 1994 – 2022 conducted in order to identify research priorities and trends. Cascading effects of climate change receives less attention than direct effects. Continuous growth present in number of papers providing more comprehensive assessment of cascading effects from climate change. Three discernable stages observed in this growth (slow, stable and most recently rapid).<br>Research encompasses natural and socio-economic systems. Food security only a prominent keyword from 2018 onwards: a lack of attention. Burst analysis generally suggests research focus has shifted from ecosystems and biodiversity to human well-being and sustainable development.<br>US largest contributor but Australia strongest international collaboration in a body of research which is otherwise dilute in cross-regional cooperation. Cooperation between developed EU countries and North American countries appeared robust. Inter-institutional collaboration followed geographical patterns, with a number of leading UK universities engaging in collaboration.
**43)** Thompson et al. (2024)	Capturing cascading consequences is required to reflect risk from climate change and natural hazards.	Evaluates the impact of including indirect impacts in climate risk assessment. Focuses on relationship between built environment and human domains. Proposes a new method to quantify indirect impacts and compares to a traditional risk assessment. New Zealand case study used. Inconsistent mapping framework/standardisation muddies overall trends in the literature: when individual papers devises their own methods it requires the reader to understand the method before being able to interpret their findings.<br>There can be significant differences in risk priority when cascading consequences are included in a climate change risk assessment, which has notable implication for planning and justifying intervention strategies.<br>Actually assessing higher-order consequences remains a research challenge.
**44)** Valencia et al. (2018)	Cascading effects from plants to soil microorganisms explain how plant species richness and simulated climate change affect soil multifunctionality.	Evaluates how plant functional structure and soil microbial diversity regulates soil responses to changes in plant species prevalence and simulated climate change. Common garden experiment completed to do so. Aims to bridge knowledge gap regarding the capacity of ecosystems to provide multiple functions under climate change. Cascading effects between plant functional traits and the abundance of soil organisms across multiple trophic levels regulate the response of soil multifunctionality to stimulated climate change.<br>Novel insights into the mechanisms underlying the effect of biodiversity and climate change on ecosystem functioning can be obtained via experiments investigating these cascading risks.
**45)** Carvalho (2024)	The cascading effects of climate change on children: extreme floods, family mobility and child well-being in Amazonia.	Reflects on the less visible dimensions of climate change risks to Children. Aims to identify impacts of climate induced flooding on children by examining how age and gender impact decisions regarding family mobility and social migration. Amazonia case study. Climate change is eroding important social networks for children and exposing them to hardship, materially and emotionally.<br>A more nuanced, integrated understanding of children's climate risk is needed.<br>Supports consensus of climate change being a threat multiplier in social and social-economic issues.
**46)** Murphy et al. (2020)	Cascading effects of climate change on plankton community structure.	Examines the isolated and combined effects of warming, acidification and reductions in phytoplankton/predator abundances through a series of factorial experiments. Warming has direct impacts on the top of plankton food webs (due to increased metabolic demands of larger species) and the overall community is shaped by top-down effects from this.<br>Other trophic groups in the food web are affected more by cascading effects than direct and mitigating the cascading effects can be equally as effective in restructuring the plankton food web as mitigating direct effects.<br>Indirect effects of climate change cannot be successfully forecasted if the interactions between different stages within a system are well understood.

Supplementary Table 3: *Continued* (Part 3).

**Authors & Year**	**Title**	**Relevant Findings**
**47)** Danylchuk et al. (2023)	Cascading effects of climate change on recreational marine flats fishes and fisheries.	Considers the combined effects of climate change (extreme temperature, precipitation, sea level rise, changes in nutrient dynamics) are having on structure and function of tropical flats ecosystem. Additionally applies existing science and literature to reveal how the changes cascade through layers of biological organisations. Emphasises the need to recognise the broader defining framework of flats in conservation and management strategies.<br>Nearshore and non-trophic flats are highly dynamic and prone to natural extremes, therefore climate change can initiate cascading effects beyond structure and function of the flats themselves (e.g. to reliant fish species or food supply shortage).<br>Impacts will operate through changes in habitat, therefore consideration of greater communities and research identifying restoration practices are needed to ensure resilience to environmental change.
**48)** Gibson et al. (2020)	Case study of the cascading effects on critical infrastructure in Torbay coastal/-pluvial flooding with climate change and 3D visualisation.	Applies an integrated and participatory approach to assess risk and enhance the climate resilience of critical infrastructure to urban flooding. Methodology is applied to a case study in Torbay. Overall aim is to provide evidence that justifies future investment in coastal defences. Flood modelling software can be used to predict or visualise direct effects of climate induced flooding, as well as assess adaptation option viability by incorporating them into models. The latter can be a basis for cost benefit analysis.<br>Combining computational modelling and 3D visualisation techniques facilitated the generation of a tool to aid stakeholder communication during the decision making process. This further helped local stakeholders and operators design and evaluate adaptation measures.<br>EU Circle methodology [21] provides a flexible framework that can assess the impacts of certain climate risks on critical infrastructure, providing a basis from which further cascading risks onto other systems may be inferred.
**49)** Semenza et al. (2022)	Climate Change and Cascading Risks from Infectious Disease.	Reviews climatic and other cascading drivers of infectious disease with projections under different climate change scenarios. Linear risk or effect assessment misinforms the following: decision and policy making; forecasting of future risk patterns; and opportunities to modify policies to reduce inherent vulnerabilities and enhance infectious disease control programs.<br>Identifying cascading risk pathways from infectious disease is needed as a first step to mitigate infectious disease threats arising from climate change.<br>Complex nature of cascading risks means that health (or any systems) need to prepare for uncertainty just as much as for predictable climate change itself.
**50)** Sullivan et al. (2024)	Cascading effects of climate change: new advances in drivers and shifts of tropical reproductive phenology.	Reviews key studies (n=10) that have enhanced phenological pattern identification, improved understanding of drivers of flowering and fruiting and linked fruiting patterns with impacts on wildlife diet, reproduction and survival. Researchers need to prioritise long-term and cross site data collection to better understand the complexity of phenology across the topics and better cope with longer-term climate variation<br>Data(climate or phenology) needs to be equivalent across different locations to make valid conclusions: wider conclusion is that quality, standardised, comparable data is vital for climate adaption at all levels.<br>Cascading impacts/trophic interaction with animals will be very diverse and specific, cascade effects could see wildlife population sizes reduced and potent species extinction.
**51)** Behie et al. (2014)	Cascading Effects of Climate Change: Do Hurricane-damaged Forests Increase Risk of Exposure to Parasites?	Tests measures of exposure and susceptibility to parasitism in animals as a result of climate change. Experimental study carried out regarding helminth transmission to black howler monkeys following a hurricane in Belize. Results of specific experimentation could suggest that climate induced disasters (such as hurricanes) could generally lead to higher levels of parasitism in animals living in affected habitats. This represents a significant cascading risk of climate change with direct implications for ecosystem interactions and disease ecology.<br>Indirect parasite transmission increases most notably following habitat disturbance induced by climate shocks.
**52)** Larsen et al. (2023)	Cascading effects of climate change and wildfire on a subarctic lake.	Presents data on a 20 year long-term lake and climate monitoring program in Alaska, in which surrounding lake catchment was burned by wildfires on two occasions in the 20 year period. Cascading effects can be wide reaching and propagate without urban infrastructure or systems and are likely to be equally detrimental to natural systems.

Supplementary Table 3: *Continued* (Part 4).

**Authors & Year**	**Title**	**Relevant Findings**
**53)** Smith-McKenna et al. (2014)	Cascading effects of feedbacks, disease, and climate change on alpine treeline dynamics.	Develops a specific, 'agent-based' model to examine interactive impacts of 'blister rust' and climate change on tree line dynamics. Interaction between vegetation, climate change and disease make ecosystems very complex processes, which in turn poses management concerns for species, systems and people dependent on key species within an ecosystem.<br>Simulation models (such as agent based models) can provide management teams with a simple view of multidimensional problems, in which cascading risks of climate change are a prime example.
**54)** Menden-Deuer et al. (2023)	Cascading, interactive, and indirect effects of climate change on aquatic communities, habitats, and ecosystems.	Addresses knowledge gap regarding indirect and interactive effects of climate change on organisms and ecosystems. Time series analysis is crucial to capturing indirect/-cascading effects, especially with the accelerating nature of climate change and the resulting effects on aquatic ecosystems. There is great need to maintain high-quality time-series data to facilitate such analysis.<br>Study of species-specific dynamics vital to deliver insights into aquatic ecosystem patterns and processes, but challenging to include within climate change investigation due to the large parameter space of ecosystem studies.<br>Further investigation needs to cover a range of scales: from small scale studies relevant for individual species to large scale studies assessing full ecosystems.
**55)** Qin et al. (2023)	Flood cascading on critical infrastructure with climate change: A spatial analysis of the extreme weather event in Xinxiang, China.	Explores the damage caused by a specific flood event in China to critical infrastructure. Provides insight into historic weather events in China in order to inform decisions made to improve flood resilience and decrease negative cascading impacts on infrastructure going forward.
**56)** Challinor et al. (2018)	Transmission of climate risks across sectors and borders.	Defines new approaches to risk assessment to account for cross-border/cross-sector climate risk transmission. Presents a typology of risk transmissions and reviews existing modelling and case-studies examining risk transmission in certain areas. Case studies show policies and institutions can reduce risks significantly through mutually beneficial cooperation.<br>Assessing the way risks transmit across sectors and subsequently coordinating policies across government departments are necessary to adapt to climate change, this can likely only be achieved through broad framings of risk.<br>Regardless of the approach, more attention should be paid to social factors and societal responses, as this is currently less prevalent or omitted in assessment.
**57)** Harrison et al. (2016)	Climate change impact modelling needs to include cross-sectoral interactions.	Assesses the differences between impacts simulated by single-sector and integrated models: 14 indicators derived from a set of impact models in single-sector or integrated framework across multiple climate and socio-economic European scenarios.<br>Single-sector models misrepresent the spatial pattern, direction and magnitude of most impacts as a result of not accounting for inter dependencies between different human or environmental systems. Policy makers should use single-sector study results with caution.<br>Discrepancies particularly pronounced for food production (particularly agricultural indicators) and water exploitation as these are highly influenced by other sectors through demand changes, land suitability and resource competition.<br>Larger discrepancies observed under different socio-economic scenarios that under different climate scenarios: future socio-economic change is highly uncertain and many environmental systems are highly sensitive to socio-economic drivers.

Supplementary Table 3: *Continued* (Part 5).

Authors & Year	Title	Relevant Findings
**58)** Hui-Min et al. (2021)	Understanding systemic risk induced by climate change.	Develops a theoretical framework to bridge the literature gap between systemic risk acknowledgment and assessment. Climate induced systemic risk initiates from GHGE and propagates through increased complexity of socio-economic systems and ongoing variance in conditions caused by climate change.<br>Systemic risk is the result of interconnection and interaction of single risks and vulnerabilities and the severity of the systemic risk is a function of these discreet risks and vulnerabilities.<br>Systemic risk assessment should focus on impact area, frequency/probability of occurring and severity and needs to take system diversity and integrated impacts into account. It generally focusses on high impact risks, of both high and low probability as these are more likely to be neglected by the government due to high mitigation cost or their scarcity deeming the investment unjustifiable.
**59)** Mathias et al. (2020)	Grounding Social Foundations for Integrated Assessment Models of Climate Change.	Presents areas of research focusing on social branches of economics in attempt to increase the integration of social processes within integrated assessment models. Additionally discusses potential implications of social integrated assessment models. Whilst Integrated Assessment Models (IAM) used to inform policy and decision making increasingly integrate biophysical and economic considerations, they lack social foundations and neglect the interaction of the social system with these existing considerations.<br>Considering social aspects in IAM offers potential to open up new perspectives regarding mitigation strategies through a greater attention to social dynamics for coping with climate change. These could facilitate a bottom-up approach connecting households' perceived role in mitigating climate change and the input received from policy.
**60)** Menk et al. (2022)	Climate Change Impact Chains: A Review of Applications, Challenges, and Opportunities for Climate Risk and Vulnerability Assessments	Reviews 47 articles that apply Impact Chains (IC) or other Climate Risk Vulnerability Assessment considering the dynamics between causes and effects regarding risks, and provide an overview of common challenges and opportunities to aid future improvements. Whilst Impact Chains supports long term climate adaptation, they need to move away from linear mapping to an "impact-web" model of risk to better account for cause-effect dynamics and understand complex processes behind risk.<br>Existing qualitative approaches often utilise stakeholders to gain specific expert knowledge on risks. Moving impact chains into a quantifiable and executable model is challenged by lack of data and understanding of systems, alongside other uncertainties.<br>It is key to communicate the relevant integration and inter-connection of effects without over-complication.
**61)** Monier et al. (2018)	Toward a consistent modelling framework to assess multi-sectoral climate impacts.	Proposes a different approach to impact modelling: integrating a geospatially resolved physical representation of impacts within a human-earth system modelling framework. Aims to build upon popular existing approaches. International co-ordination or standardisation regarding scenario modelling does not necessarily facilitate effective policy responses and may yield inconsistent estimation or forecasts.<br>Instead, it is suggested to shift to using integrated modelling framework that is self-consistent in order to account for regional variation and negate the need for complex internationally coordinated modelling efforts.
**62)** Rising et al. (2022)	The missing risks of climate change.	Presents an overview of the complex and often unquantifiable economic risks associated with climate change and present an ontology explaining the lack of evolution of these. Economic effects of complex risks of climate change are difficult to quantify, creating unknowns or 'missing' risks as a result of: delayed information sharing across disciplines, spatial and temporal differences regarding climate impacts, indirect risks caused feedback/interaction which is not accounted for and other uncertainties regarding knowledge and risks already identified.<br>Both model-based quantification and qualitative assessment will be important in identifying and dealing with these missing risks. In particular, enhanced interdisciplinary collaboration and deeper consideration of uncertainty is required to inform policy makers and the general public on less obvious risks.<br>Paper offers and validates their own framework to find and assess less obvious risks.

Supplementary Table 3: *Continued* (Part 6).

**Authors & Year**	**Title**	**Relevant Findings**
**63)** Simpson et al. (2021)	A framework for complex climate change risk assessment.	Presents a framework to assess three categories of climate risks, focusing on the interaction between risks and their drivers in order to address lack of a holistic framework assessing complex and cascading risks. Risks can arise from direct climate impacts and their propagation through systems, but also from other responses made to combat climate change, with the latter are often overlooked or assessed separately. Thus the integration of these responses into climate change risk assessment deepens understanding and increases the relevance of climate resilience for policy makers.<br>Further development of new approaches to risk assessment and decision support that encompasses complexities of interaction are necessary to keep societies safe.
**64)** Yokohata et al. (2019)	Visualizing the Interconnections Among Climate Risks.	Fills the literature gap on the exploration of interconnections between climate risks by developing a method for visualising climate risks and interconnections using a literature survey. Climate risk diagrams developed using existing techniques available for mapping complex data, such as ’network maps’ and ’network flowcharts’, may serve as a useful tool to enhance public understanding on cascading effects of climate change.<br>A major challenge to visualising cascading effects of climate change is achieving a balance in the diagram complexity (given the complex nature of cascading risks), with readability. This often requires prioritisation and selection of certain risk interconnections, which is aided by a systematic approach.<br>Visualisation of cascades through static figures as produced in this work is difficult. Interactive visualisation tools shown to have a strong effect on perceived reality of climate change. As such, developing interactive aspects to these visualisations may improve usefulness when acquiring public engagement.

Supplementary Table 3: *Continued* (Part 7).

References

[1] CRADOCK-HENRY, N. A. et al. Elaborating a systems methodology for cascading climate change impacts and implications. *MethodsX*, Elsevier, v. 7, p. 100893, 2020.

[2] STEELE, C. et al. Cascading impacts of climate change on southwestern US cropland agriculture. *Climatic Change*, Springer, v. 148, p. 437–450, 2018.

[3] PAGE, M. J. et al. The PRISMA 2020 statement: An updated guideline for reporting systematic reviews. *BMJ*, British Medical Journal Publishing Group, v. 372, p. n71, 2021.

[4] WHEELER, R.; LOBLEY, M. Managing extreme weather and climate change in UK agriculture: Impacts, attitudes and action among farmers and stakeholders. *Climate Risk Management*, Elsevier, v. 32, p. 100313, 2021.

[5] LIAQAT, W. et al. Climate change in relation to agriculture: A review. *Spanish Journal of Agricultural Research*, v. 20, n. 2, 2022.

[6] MALHI, G. S.; KAUR, M.; KAUSHIK, P. Impact of climate change on agriculture and its mitigation strategies: A review. *Sustainability*, MDPI, v. 13, n. 3, p. 1318, 2021.

[7] ANDERSON, R.; BAYER, P. E.; EDWARDS, D. Climate change and the need for agricultural adaptation. *Current Opinion in Plant Biology*, Elsevier, v. 56, p. 197–202, 2020.

[8] WREFORD, A.; TOPP, C. F. Impacts of climate change on livestock and possible adaptations: A case study of the United Kingdom. *Agricultural Systems*, Elsevier, v. 178, p. 102737, 2020.

[9] CHENG, M.; MCCARL, B.; FEI, C. Climate change and livestock production: a literature review. *Atmosphere*, MDPI, v. 13, n. 1, p. 140, 2022.

[10] ROJAS-DOWNING, M. M. et al. Climate change and livestock: Impacts, adaptation, and mitigation. *Climate Risk Management*, Elsevier, v. 16, p. 145–163, 2017.

[11] GAUGHAN, J.; CAWDELL-SMITH, A. Impact of climate change on livestock production and reproduction. *Climate Change Impact on Livestock: Adaptation and Mitigation*, Springer, p. 51–60, 2015.

[12] CALLAWAY, R. et al. Review of climate change impacts on marine aquaculture in the UK and Ireland. *Aquatic Conservation: Marine and Freshwater Ecosystems*, Wiley Online Library, v. 22, n. 3, p. 389–421, 2012.

[13] DABBADIE, L. et al. Effects of climate change on aquaculture: drivers, impacts and policies. *Impacts of Climate Change on Fisheries and Aquaculture*, p. 449, 2019.

[14] BARANGE, M. et al. Impacts of climate change on fisheries and aquaculture. *United Nations' Food and Agriculture Organization*, v. 12, n. 4, p. 628–635, 2018.

[15] LOWE, J. A. et al. UKCP18 science overview report. *Met Office Hadley Centre: Exeter, UK*, p. 1–73, 2018.

[16] FROST, M. et al. Impacts of climate change on fish, fisheries and aquaculture. *Aquatic Conservation: Marine and Freshwater Ecosystems*, John Wiley & Sons, Ltd Chichester, UK, v. 22, n. 3, p. 331–336, 2012.

[17] UK Government. *UK Climate Change Risk Assessment 2022*. 2022. ⟨https://assets.publishing.service.gov.uk/media/61e54d8f8fa8f505985ef3c7/climate-change-risk-assessment-2022.pdf⟩. Accessed: 2024-08-10.

[18] Ventana Systems, Inc. *Vensim Software*. 2024. ⟨https://vensim.com/software/⟩. Accessed: 2024-07-31.

[19] LEVY, S. ECDC Vibrio Map Viewer: Tracking the whereabouts of pathogenic species. *Environmental Health Perspectives*, v. 126, n. 3, p. 034003, 2018.

[20] World Health Organization. *One Health*. 2024. ⟨https://www.who.int/news-room/fact-sheets/detail/one-health⟩. Accessed: 2024-07-31.

[21] GUZE, S.; KOŁOWROCKI, K. EU-CIRCLE: A pan-European framework for strengthening critical infrastructure resilience to climate change Project taxonomy and methodology: Resilience terminology and methodology. *Journal of Polish Safety and Reliability Association*, v. 8, 2017.
